# Simulated Microgravity and 3D Culture Enhance Induction, Viability, Proliferation and Differentiation of Cardiac Progenitors from Human Pluripotent Stem Cells

**DOI:** 10.1038/srep30956

**Published:** 2016-08-05

**Authors:** Rajneesh Jha, Qingling Wu, Monalisa Singh, Marcela K. Preininger, Pengcheng Han, Gouliang Ding, Hee Cheol Cho, Hanjoong Jo, Kevin O. Maher, Mary B. Wagner, Chunhui Xu

**Affiliations:** 1 Division of Pediatric Cardiology, Department of Pediatrics, Emory University School of Medicine and Children’s Healthcare of Atlanta, Atlanta, GA, USA; 2 Wallace H. Coulter Department of Biomedical Engineering, Georgia Institute of Technology and Emory University, Atlanta, GA, USA; 3 Division of Cardiology, Department of Medicine, Emory University School of Medicine, Atlanta, GA, USA

## Abstract

Efficient generation of cardiomyocytes from human pluripotent stem cells is critical for their regenerative applications. Microgravity and 3D culture can profoundly modulate cell proliferation and survival. Here, we engineered microscale progenitor cardiac spheres from human pluripotent stem cells and exposed the spheres to simulated microgravity using a random positioning machine for 3 days during their differentiation to cardiomyocytes. This process resulted in the production of highly enriched cardiomyocytes (99% purity) with high viability (90%) and expected functional properties, with a 1.5 to 4-fold higher yield of cardiomyocytes from each undifferentiated stem cell as compared with 3D-standard gravity culture. Increased induction, proliferation and viability of cardiac progenitors as well as up-regulation of genes associated with proliferation and survival at the early stage of differentiation were observed in the 3D culture under simulated microgravity. Therefore, a combination of 3D culture and simulated microgravity can be used to efficiently generate highly enriched cardiomyocytes.

Heart disease is a major health concern, claiming more lives each year than any other diseases^1^. Cardiomyocytes (CMs) derived from human pluripotent stem cells (hPSCs) could provide an unlimited supply of cells to replenish the lost cardiac muscle. In preclinical studies, hPSC-CMs and hPSC-cardiac progenitors have been found to prevent progression of heart failure in animal models^2^,^3^,^4^,^5^,^6^,^7^,^8^,^9^. It is estimated that ~10^9^ CMs are needed to repair a failing human heart, and graft survival is challenging—for example, in over 90% of transplanted hPSC-CMs die even with pro-survival pretreatment in a nonhuman primate model^10^. Therefore, to fully realize the potentials of hPSCs, efficient and robust generation of large quantities of CMs is critical. CM differentiation requires specific induction of the transition from stem cells to cardiac progenitors with growth factors^2^,^11^, small molecules^12^,^13^, signals from endodermal environment^14^,^15^ and matrix proteins^16^. It is also conceivable that promoting proliferation of cardiac progenitors and increasing cell viability during *in vitro* differentiation could increase the CM yield and improve graft survival.

3D culture and microgravity, a condition in which objects appear to be weightless, can profoundly modulate cell proliferation and survival. 3D culture allows cells to self-organize by aggregation and facilitate spatially unrestricted interactions between cells and their surroundings, circumventing the disadvantages of 2D culture that limit cell-cell signaling and restrict cell growth in an artificial environment^17^. Consequently, incorporating 3D culture during the transition from cardiac progenitors to CMs may facilitate the proliferation and survival of cardiac progenitors. In addition, 3D culture has advantages for scale up production of hPSCs and their derivatives^18^,^19^,^20^. Microgravity is also known to modulate cell proliferation and survival^21^. For example, simulated microgravity potentiates the proliferation of bone marrow-derived human mesenchymal stem cells^22^ and adipose-derived stem cells^23^. Bioreactors have been designed to simulate aspects of microgravity and weightless environment during spaceflight and have been utilized to culture many cell types including stem cells, osteoblasts and cancer cells^24^,^25^,^26^. In these systems, cells can form complex multicellular aggregates or organoids and can be maintained for days and months in a gentle, low-shear and low-turbulence environment with sufficient oxygenation and effective mass transfer of nutrient and waste.

In this study, we have examined whether 3D tissue engineering of cardiac progenitors in combination with simulated microgravity could improve the efficiency of CM generation from hPSCs. We generated cardiac progenitors from hPSCs, engineered them into multicellular 3D progenitor cardiac spheres through controlled aggregation, and then examined the impact of 3D culture and simulated microgravity on CM purity, viability and yield. In addition, we analyzed CM induction, proliferation, cell survival and molecular changes in early-stage cardiac cells in an effort to gain possible mechanistic insights of the effect of 3D culture and simulated microgravity on differentiation.

## Results

### Suspension culture of progenitor cardiac spheres and simulated microgravity increase cell viability and CM yield

We initially characterized starting materials of hPSCs and evaluated the efficiency of cardiac induction. At day 0, the culture displayed sheet-like morphology and contained >95% TRA1-60^pos^ stem cells (Fig. S1A). At day 4, cells lost typical stem cell morphology (Fig. S1B) and >90% of them expressed a cardiac mesoderm marker, *MESP1,* which is typically up-regulated at days 4 to 5^27^.

To generate 3D cell aggregates of cardiac progenitors using a microscale technique, day 4 cells were dissociated and force-aggregated in a microwell plate at three different densities: 500, 1500 and 2500 cells/microwell. After 24 h, sphere-shaped cell aggregates, named progenitor cardiac spheres, were generated from all cultures (Fig. S1C). After culture in suspension, cardiac spheres from cultures seeded at densities of 1500 and 2500 cells/microwell were more compact than those from cultures seeded at densities of 500 cells/microwell (Fig. S1C). Similar results were observed when progenitor cardiac spheres were generated from day 6 cells. At day 20, almost all cardiac spheres showed spontaneous beating, and α-actinin, a CM-associated protein, was detected in ~62%, ~87% and ~82% of the cells in cultures with seeding densities of 500 cells/microwell, 1500 cells/microwell and 2500 cells/microwell, respectively (Fig. S1D). Thus, seeding density of 1500 cells/microwell was selected for subsequent experiments.

**Figure 1:**
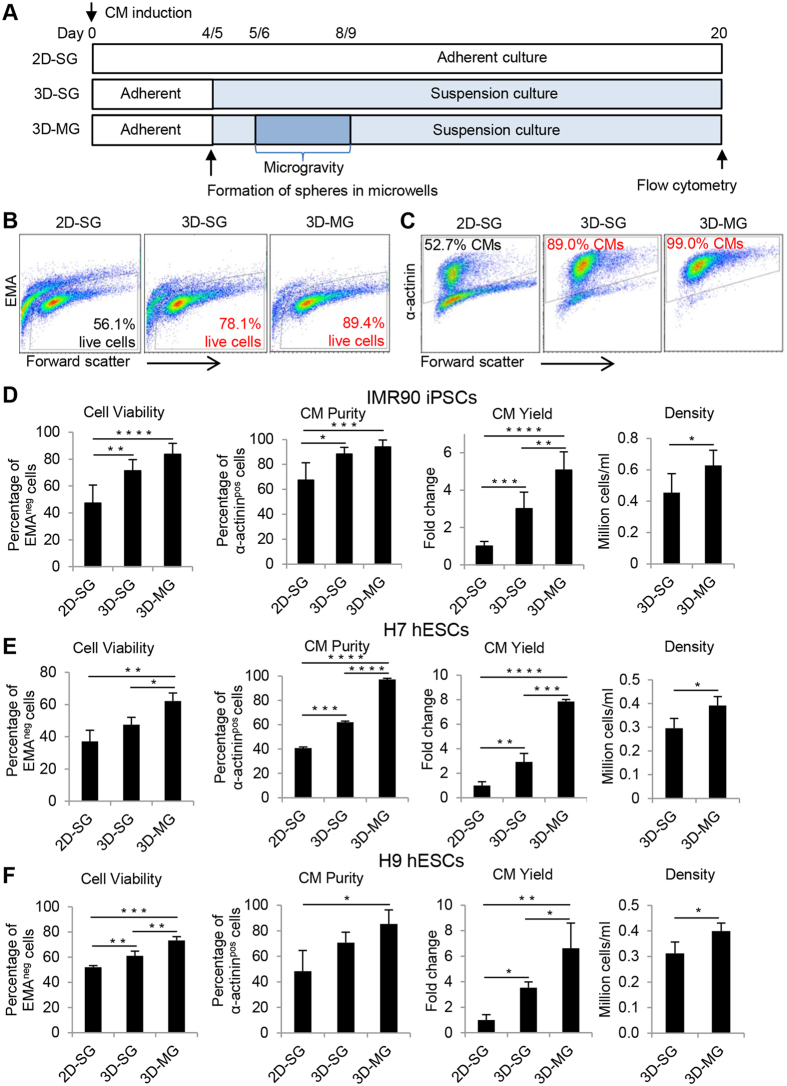
Suspension culture of progenitor cardiac spheres and simulated microgravity increase cell viability and CM yield. (**A**) Experimental design. hPSCs were induced to differentiate into CMs at day 0 using growth factors or small molecules. At day 4 or 5, cells were dissociated and forced to aggregate into progenitor cardiac spheres. The progenitor cardiac spheres were cultured under simulated microgravity at days 5–8 or 6–9 and maintained under standard gravity until day 20 (designated as 3D-MG). Parallel 2D and 3D cultures were maintained under standard gravity throughout, designated as 2D-SG and 3D-SG, respectively. At day 20, cells were analyzed for cell viability, CM purity, yield and density. (**B**) Representative flow cytometry analysis. Cell viability was analyzed by EMA staining and EMA negative cells were identified as live cells. Purity of CMs was analyzed by intracellular staining of α-actinin, a CM-associated marker. (**C**) Summary of IMR90-iPSC differentiated cell viability, CM purity, cell yield and density. (**D**) Summary of H7 hESC differentiated cell viability, CM purity, cell yield and density. (**E**) Summary of H9 hESC differentiated cell viability, CM purity, CM yield and density. The CM yield was defined as the number of viable CMs generated from one undifferentiated stem cell. Data are presented as mean ± SD of 3–6 biological samples for each culture condition. *P < 0.05; **P < 0.01; ***P < 0.001, ****P < 0.0001.

To examine the effect of simulated microgravity and 3D culture on CM differentiation (Fig. 1A), progenitor cardiac spheres were generated from day 4 IMR-90 iPSCs induced by activin A and BMP4 (Fig. S1 and S3A), transferred to OptiCell disks and maintained under simulated microgravity using a random positioning machine (RPM) (Fig. S2B and S2C). After the exposure to simulated microgravity for 3 days (the duration was optimized – Fig. S2), the culture, designated as 3D-MG, was transferred to standard gravity at day 8. Parallel cultures of 3D spheres in suspension under standard gravity (3D-SG) and 2D cultures under standard gravity (2D-SG) were also maintained for comparison. Overall cell morphology of 3D-MG was similar to that of 3D-SG; the spheres were similar in size 24 h after aggregation and became irregular in shape at later time points, possibly due to random budding of cell growth (Fig. S3B, S3C and S3D). Typically, contracting cells were observed by differentiation days 8–10 in both 2D and 3D cultures. At day 20, almost all spheres in 3D-SG and 3D-MG showed spontaneous beating, while only some beating areas were observed in 2D-SG (Movies S1,2 to S3). The 3D-SG and 3D-MG cultures at day 20 contained up to 99% α-actinin^pos^ cells, which was higher than observed in 2D-SG (Fig. 1B–D, P < 0.001 to 0.05).

Cell viability assayed by ethidium monoazide (EMA) staining in a representative experiment showed that ~90% and 78% of the cells from 3D-MG and 3D-SG, respectively, were viable (EMA negative) whereas only ~56% of the cells from 2D-SG were viable (Fig. 1B). Analysis of 5 to 6 cultures per condition indicated that 3D-SG and 3D-MG contained a higher percentage of viable cells than 2D-SG (Fig. 1D, P < 0.01 and <0.001, respectively). To exclude the possibility that these differences might be caused by cell dissociation of tightly packed 2D or 3D culture, we also performed Live/Dead staining of intact cultures. As shown in Fig. S3D, 3D-MG and 3D-SG had fewer dead cells than did 2D-SG, which was consistent with the observation by flow cytometry assay of post-dissociated cells (Fig. 1).

The CM yield was 1.5-fold and 5-fold higher in 3D-MG than the 3D-SG and 2D-SG, respectively (Fig. 1D and Fig. S4, P < 0.01 and <0.0001, respectively). Cell density was also higher in 3D-MG (6.3 ± 1.0 × 10^5^ cells/ml) than 3D-SG (4.5 ± 1.2 × 10^5^ cells/ml and P < 0.05).

We also examined the effect of simulated microgravity and 3D culture on CM differentiation from H7 and H9 human embryonic stem cells (hESCs) (Fig. 1E,F). Cells were induced for CM differentiation by small molecules targeting the Wnt signaling^13^ and exposed to simulated microgravity from days 6 to 9. At day 20, the proportions of viable cells were higher in 3D-MG than 3D-SG and 2D-SG (P < 0.05 and<0.01, respectively). CM purity of H7-derived cells as detected by α-actinin^pos^ cells was ~97% in 3D-MG while only ~62% and ~41% CMs were detected in 3D-SG and 2D-MG, respectively. CM purity of H9-derived cells was ~85% in 3D-MG while only ~71% and ~48% CMs in 3D-SG and 2D-MG, respectively. The CM yield was 3 to 4-fold and 7 to 8-fold higher in 3D-MG than 3D-SG and 2D-SG, respectively (Fig. 1E,F and Fig. S4). Cell density was also higher in 3D-MG than 3D-SG (P < 0.05). These results indicated that simulated microgravity and 3D culture significantly improved cell viability, CM purity and yield in H7 and H9 cells.

We then examined the effect of simulated microgravity and 3D culture on endothelial cell differentiation since mesoderm stage MESP1^pos^ cells could give rise to endothelial cells. However, at differentiation day 20, very few cells (<2%) were positive for endothelial cell makers, CD31, vascular endothelial cadherin (VE-cadherin) in 3 culture conditions (2D-SG, 3D-SG and 3D-MG) examined (Fig. S6).

To examine if improved CM differentiation in 3D culture was due to dissociation of cardiac progenitor cells and if low cell yield in 2D culture was due to the limitation in space for cell expansion, day 4 cells were dissociated and re-seeded at 3 densities (undiluted, diluted 2 times and 4 times) in 2D culture and CM differentiation efficiency was compared at day 20 with parallel intact cultures. As shown in Fig. S5, the levels in cell viability, CM purity and yield from the dissociated 2D cultures were similar to the intact 2D cultures, suggesting that cell dissociation and providing additional space to cells in 2D culture were not sufficient to improve CM yield.

These observations showed that 3D cultures produced CMs with greater purity and higher yield than 2D culture and that exposure of 3D progenitor cardiac spheres to simulated microgravity improved CM yield even further.

### Characterization of CMs derived from progenitor cardiac spheres expanded under standard gravity and simulated microgravity

**Figure 2:**
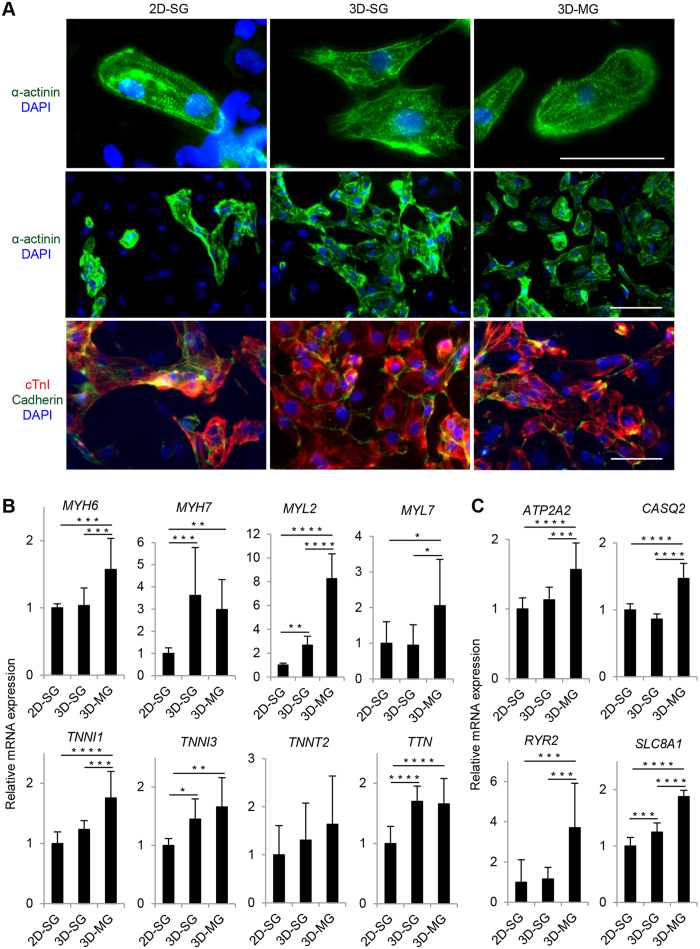
Cellular and molecular features of CMs derived from progenitor cardiac spheres expanded under standard gravity and simulated microgravity. (**A**) Immunocytochemistry analysis of the cardiac structural proteins α-actinin and cTnI and the adhesion molecule cadherin in cells harvested at day 20. Nuclei were stained with DAPI. Scale bars = 50 μm. (**B**,**C**) Expression of genes encoding structural proteins and calcium handling proteins in these cells determined by qRT-PCR. Data are presented as mean ± SD of 4 biological replicates x 3 reactions/sample. *P < 0.05; **P < 0.01; ***P < 0.001; ****P < 0.0001.

We next examined cellular and molecular features of the CMs from 3D-SG and 3D-MG. As shown in Fig. 2A, all cultures contained cells that were positive for CM-associated markers, including structural proteins, α-actinin and cardiac troponin I (cTnI), and cell-cell adhesion molecule, cadherin. Overall, 3D-SG and 3D-MG showed more cells positive for these CM-associated markers than did 2D-SG, which is consistent with increased CM purity in 3D-SG and 3D-MG than 2D-SG.

We also examined the expression of genes encoding CM structural proteins (Fig. 2B) and calcium handling proteins (Fig. 2C). Of the 12 genes examined, 11 (*MYH6*, *MYH7*, *MYL2*, *MYL7*, *TNNI1*, *TNNI3*, *TNN, ATP2A2, CASQ2, RYR2* and *SLC8A1*) were detected at higher levels in 3D-MG than 2D-SG (P < 0.0001 to 0.05). In addition, 8 (*MYH6, MYL2*, *MYL7*, *TNNI1*, *ATP2A2, CASQ2, RYR2* and *SLC8A1*) were detected at higher levels in 3D-MG than 3D-SG (P < 0.001 to 0.05), and none was lower in 3D-MG than 3D-SD.

**Figure 3:**
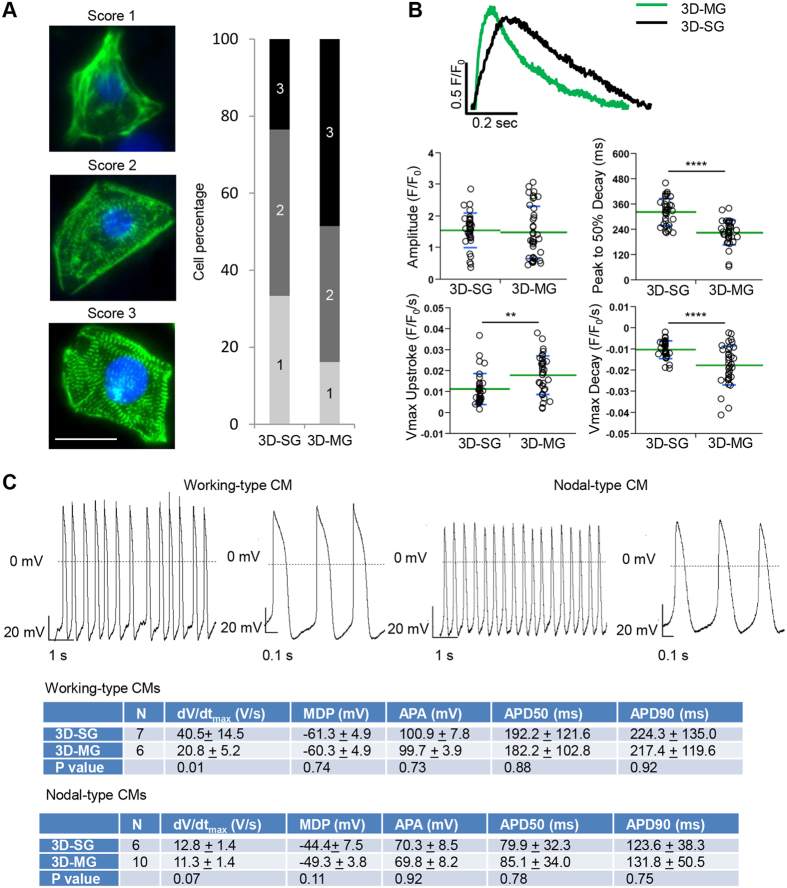
Structural and functional characterization of hPSC-CMs cultured in 2D-SG, 3D-SG and 3D-MG conditions. (**A**) Structural analysis of hPSC-CMs. Cells were dissociated, replated and stained for sarcomeric α-actinin (green) and DAPI (blue). Overall appearance of myofibrillar structure was categorized into 3 different levels: Score 1 cells are α-actinin^pos^ but without clear sarcomeric striations; Score 2 cells have diffuse punctate staining pattern and some patterned striations in partial cell area; and Score 3 cells have highly organized and well-defined myofibrillar structure with distinct paralleled bands of z-discs distributed throughout the cell area. Percentage of the cells by the scores was generated by counting 102 and 136 cells for 3D-SG and 3D-MG, respectively. Note that 3D-MG produced CMs with higher levels structural maturation. Scale bar = 25 μm. (**B**) Calcium transients of hPSC-CMs cultured in 3D-SG and 3D-MG conditions. Representative traces of hPSC-CMs field-stimulated at 1 Hz were acquired by optical fluorescence imaging. Measurements of calcium transients are presented as mean ± SD of n = 37 and 40 line scans for 3D-SG and 3D-MG culture conditions, respectively. **P < 0.01; ****P < 0.0001. (**C**) Representative patch clamp recording and summary of action potential parameters. Electrophysiological parameters measured: N, cell number; dV/dtmax, maximum action potential upstroke velocity; MDP, maximum diastolic potential; APA, action potential amplitude; APD50, action potential duration at 50% of repolarization; APD90, action potential duration at 90% of repolarization. Data are presented as mean ± SD.

Under higher magnification, sarcomeric structures were observed in a subset of CMs in all cultures stained with α-actinin, confirming the characteristics of CMs. To examine the effect of simulated microgravity on sarcomeric organization, we carried out immunostaining of α-actinin, a marker for myofibrillar Z-discs. Typically hPSC-CM cultures contain CMs with different levels of sarcomeric maturation. We evaluated these cells for their overall appearance of myofibrillar structure and categorize them into 3 different levels as we and others described previously^28^,^29^: Score 1 cells are α-actinin^pos^ but without clear sarcomeric striations; Score 2 cells have diffuse punctate staining pattern and some patterned striations in partial cell area; and Score 3 cells have highly organized and well-defined myofibrillar structure with distinct paralleled bands of z-discs distributed throughout the cell area. Compared with 3D cardiac spheres expanded under standard gravity, 3D cardiac spheres exposed to simulated microgravity produced CMs with higher levels of structural maturation based on the levels of sarcomeric striations (Fig. 3A), although T tubules were not detectable in cells from any conditions.

We next investigated the effect of simulated microgravity on Ca^2+^ transients by confocal line-scan recordings of live 3D-SG and 3D-MG cells (Fig. 3B). There were no significant differences in fluorescent amplitude (F/F_0_) between the two groups. However, the maximal upstroke and decay velocities were ~38% (P < 0.01) and ~44% (P < 0.0001) faster in 3D-MG CMs compared to 3D-SG CMs, respectively. Furthermore, the time from the peak to 50% decay was ~38% shorter (P < 0.0001) in 3D-MG CMs compared to 3D-SG CMs, demonstrating an enhanced rate of Ca^2+^ reuptake, a feature associated with more mature CMs^30^. The calcium imaging data is consistent with the increased expression of *ATP2A2* (*SERCA2a*) and *SLC8A1* (*NCX*) in 3D-MG compared with 3D-SG (Fig. 2C) which suggested that 3D-MG had improved calcium handling gene expression because CM purity of 3D-MG was similar to that of 3D-SG.

Action potential parameters including depolarization velocity (dV/dtmax), resting membrane potential (MDP), action potential amplitude (APA) and action potential duration (APD) of CMs from 3D-MG detected by patch-clamp were not significantly different from those from 3D-SG (Fig. 3C; P > 0.05 for all parameters except that the dV/dtmax of 3D-SG CMs was faster than that of 3D-MG CMs.)

**Figure 4:**
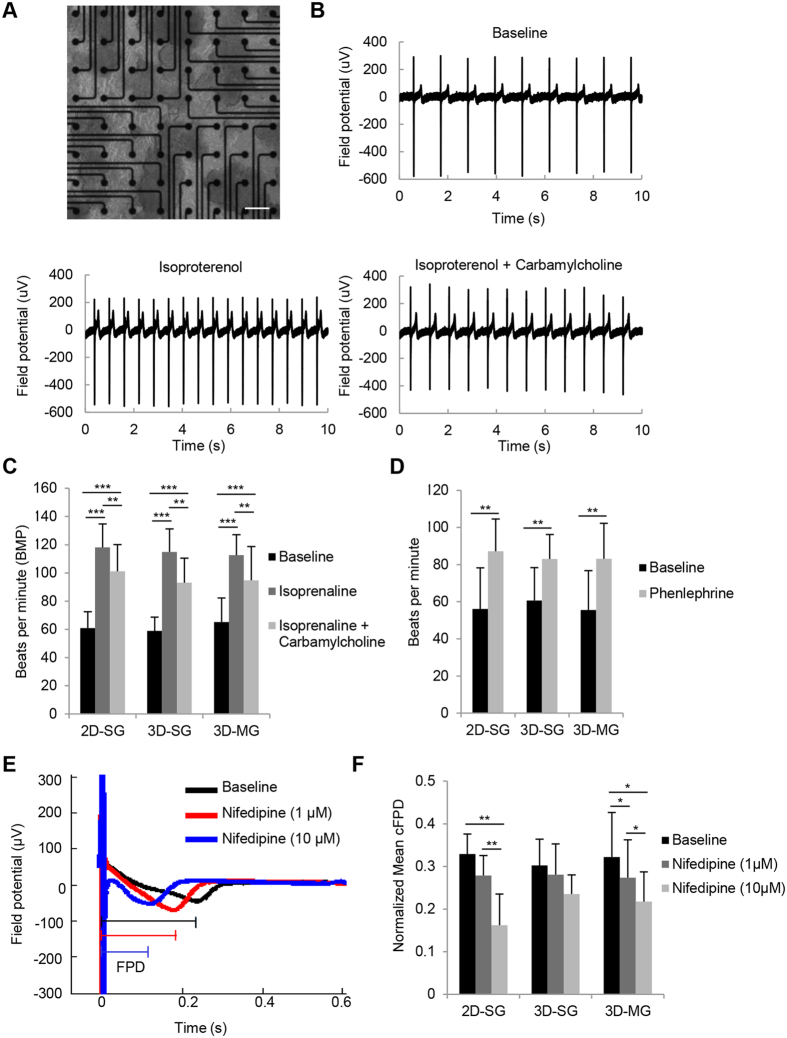
MEA recordings of hPSC-CMs cultured in 2D-SG, 3D-SG and 3D-MG conditions. (**A**) A representative image of MEA chamber seeded with cardiospheres. Extracellular recordings from differentiated culture harvested at day 20 without treatment (baseline) and after the treatment with indicated pharmacological agents. Scale bars = 100 μm. (**B**) Representative MEA recordings of cells without treatment (baseline), of cells treated alone with isoproterenol as indicated, and of cells first treated with isoproterenol alone followed by co-treatment of carbamylcholine (isoproterenol + carbamylcholine). (**C**,**D**) Response of cell beating to the indicated pharmacological treatments. n = 4 biological samples. (**E**) Representative MEA recording before and after cells were treated with nifedipine at final concentrations of 1 and 10 μM. (**F**) Normalized mean cFPD of cells before and after the nifedipine treatment. n = 3–4 biological samples. All data are presented as mean ± SD. *P < 0.05; **P < 0.01; ***P < 0.001.

Using microelectrode array (MEA) recordings, we investigated pharmacological responses of CMs from the three cultures (Fig. 4A). When the cells were treated with β-adrenergic agonist isoproterenol, all cultures showed an increase in spontaneous beating rate (Fig. 4B,C, P < 0.001); the increased beating was attenuated when cells were further treated with carbamylcholine, a cholinergic agonist, (Fig. 4B,C, P < 0.001 to 0.01). When the cells were treated with phenylephrine, an α1-adrenergic receptor agonist, beating rates in all cultures increased (Fig. 4D, P < 0.01). In addition, when cells were treated with nifedipine, a calcium channel blocker, normalized mean corrected field potential durations (cFPD) of the cells in all 3 cultures decreased (Fig. 4E,F; P < 0.01 to 0.05).

These data suggest that CMs generated from 3D-SG and 3D-MG have similar electrophysiological properties and pharmacological responses.

### Simulated microgravity and 3D culture promote the induction of cardiac progenitors and CM differentiation

**Figure 5:**
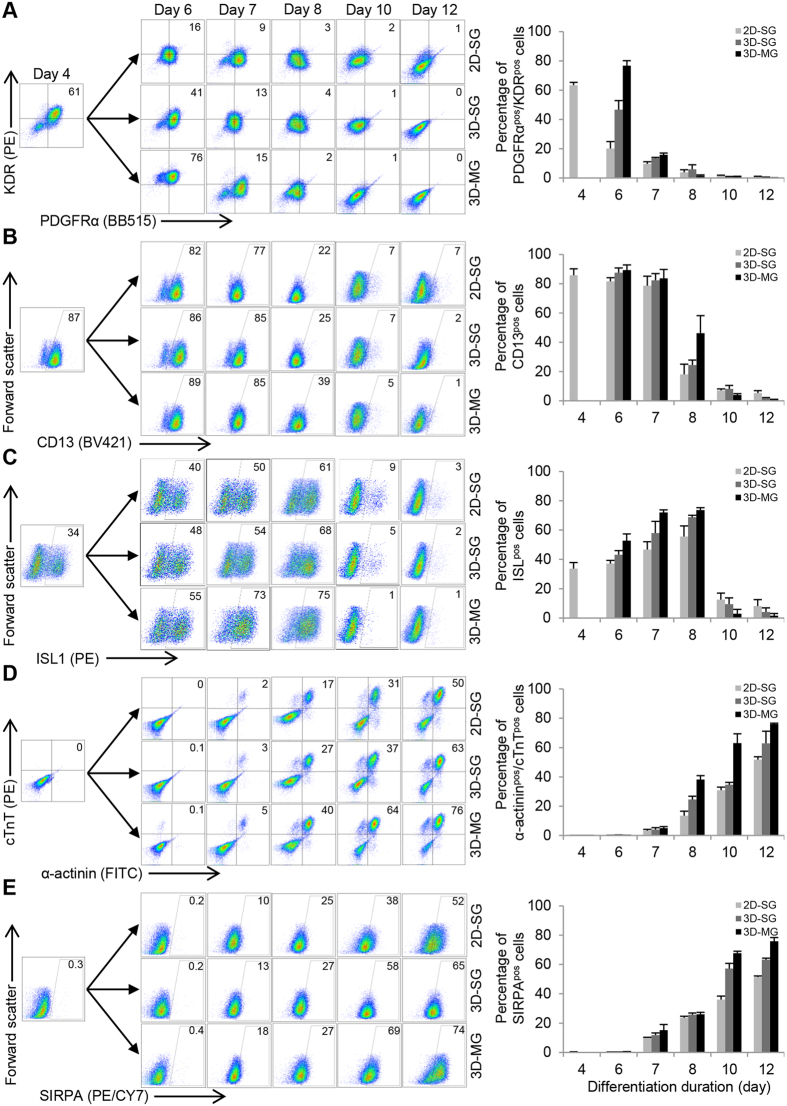
Simulated microgravity and 3D culture promote the induction of cardiac progenitors and CM differentiation. Flow cytometry analysis of cells exposed to microgravity (3D-MG) and standard gravity (2D-SG and 3D-SG) at various time points for their expression of markers associated with (1) cardiac mesoderm, (**A**) KDR/PDGFRα and (**B**) CD13; (2) cardiac progenitors, (**C**) ISL1; and (3) cardiomyocytes, (**D**) SIRPA and (**E**) cTnT/α-actinin. Data are presented as representative flow cytometry analysis and summary based on mean ± SD of 3 biological replicates.

To examine the effect of simulated microgravity and 3D culture on the induction of cardiac progenitors and CM differentiation, we analyzed the expression of markers associated with cardiac progenitors and CMs at various time points from differentiation days 4 to 12. At day 4, ~61% of the population were positive for KDR and PDGFRα, markers associated with cardiac mesoderm induction^11^. At day 6, KDR^pos^/PDGFRα^pos^ cells increased to ~76% in 3D-MG, but decreased to ~16% and ~41% in 2D-SG and 3D-SG, respectively (Fig. 5A). At day 8, 3D-MG cells retained the expression of CD13, a cardiac mesoderm marker^31^,^32^ at higher levels compared with cells under standard gravity (Fig. 5B). The expression of ISL1, a marker for cardiac progenitors^33^, increased overtime with a peak at day 8 to ~75% in 3D-MG as compared with ~61% and ~68% in 2D-SG and 3D-SG, respectively (Fig. 5C). These results suggest that simulated microgravity and 3D culture increase the induction of cardiac progenitors.

We next examined if these cardiac progenitors led to robust CM differentiation (Fig. 5D,E). At day 12, the proportions of cells that were positive for SIRPA, a surface CM marker, increased to ~74% in 3D-MG compared with ~52% and ~65% in 2D-SG and 3D-SG, respectively. Similarly, the proportions of cTnT^pos^/α-actinin^pos^ cells were the highest in 3D-MG at day 12. These result suggest that simulated microgravity and 3D culture promote CM differentiation.

### Simulated microgravity and 3D culture promote the proliferation of cardiac progenitors

**Figure 6:**
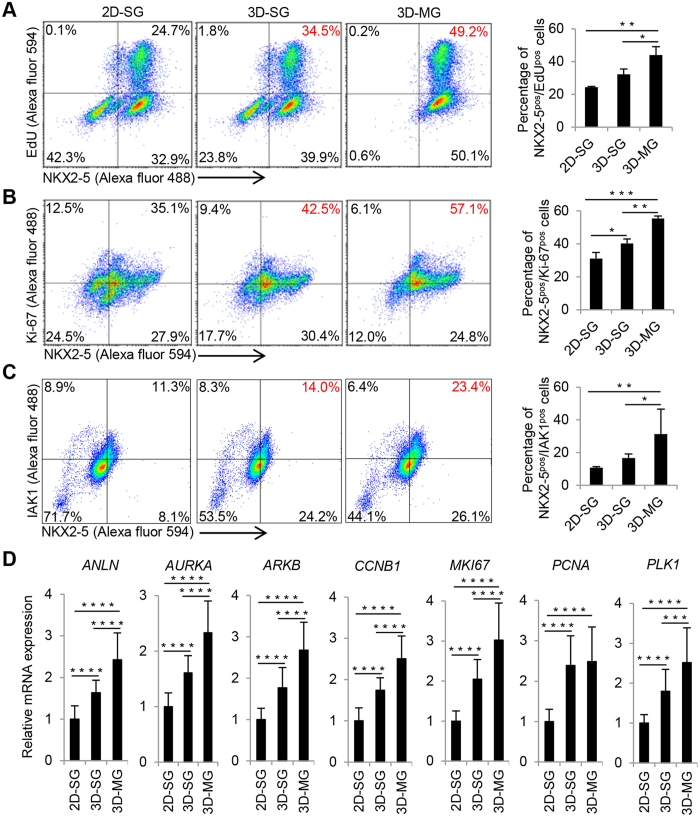
Simulated microgravity and 3D culture increase the proliferation of cardiac progenitors. (**A)** Representative images, flow cytometry analysis and summary of cells at differentiation day 10 that were co-stained with EdU and an antibody against NKX2-5, a marker for cardiac progenitors. Scale bars = 100 μm. The quantitative summary was based on flow cytometry analysis. Data are presented as mean ± SD of 3 biological replicates. Scale bar = 100 μm. (**B**) Similar analyses to (**A**) except that Ki-67 instead of EdU was co-stained with NKX2-5. (**C**) Similar analyses to (**A**) except that IAK1 instead of EdU was co-stained with NKX2-5. (**D**) Relative expression levels of genes associated with proliferation and cell cycle in cells at differentiation day 8 according to qRT-PCR. Data are presented as mean ± SD of 8 biological replicates x 3 reactions/sample. *P < 0.05; **P < 0.01; ***P < 0.001; ****P < 0.0001.

To determine if there was increased proliferation of cardiac progenitors in 3D-SG and 3D-MG at the early stage (which may contribute to the higher cell yield and viability in the 3D cultures at late stages), we examined the ability of the cells to incorporate EdU as an indicator of proliferation. Among the NKX2-5^pos^ progenitors at day 10, a subset of them was EdU^pos^ (Fig. S7A). By flow cytometry, 24.4%, 32.3% and 44.0% were identified as EdU^pos^/NKX2-5^pos^ cells in 2D-SG, 3D-SG and 3D-MG, respectively (Fig. 6A). The number of EdU^pos^/NKX2-5^pos^ cells was higher in 3D-MG than 3D-SG and 2D-SG (P < 0.05 and <0.01 respectively).

We also found that a subset of the NKX2-5^pos^ cells at day 10 expressed Ki-67, a marker for cells at active phases of the cell cycle (Fig. S7B). By flow cytometry analysis, ~31%, ~40% and ~55% of the cells were NKX2-5^pos^/Ki-67^pos^ in 2D-SG, 3D-SG and 3D-MG, respectively (Fig. 6B). The proportions of NKX2-5^pos^/Ki-67^pos^ cells were higher in 3D-SG and 3D-MG than 2D-SG (P < 0.05 and <0.001, respectively), and also higher in 3D-MG than 3D-SG (P < 0.01). Similarly, a subset of the NKX2-5^pos^ cells at day 8 expressed IAK1 (aurora-A kinase), a key regulator in the control of cell proliferation (Fig. 6C). The proportions of NKX2-5^pos^/IAK1^pos^ cells were higher in 3D-MG than 2D-SG and 3D-SG (P < 0.001 and <0.05, respectively).

To further investigate the effect of 3D culture and simulated microgravity on cell proliferation, we also examined the expression of genes associated with proliferation and cell cycle at day 8 by qRT-PCR. Higher expression levels of genes related to cell proliferation (*MKI67* and *PCNA*) and cell cycle (*ANLN*, *AURKA*, *AURKB*, *CCNB1* and *PLK1*) were detected in 3D-SG and 3D-MG than 2D-SG (P < 0.0001, Fig. 6C). Furthermore, expression levels of genes associated with cell cycle and proliferation (*ANLN*, *AURKA*, *AURKB*, *CCNB1, MKI67,* and *PLK1;* 6 out of 7 examined) were higher in 3D-MG than 3D-SG (P < 0.0001 to 0.001).

### Simulated microgravity and 3D culture increase cell survival of cardiac progenitors

**Figure 7:**
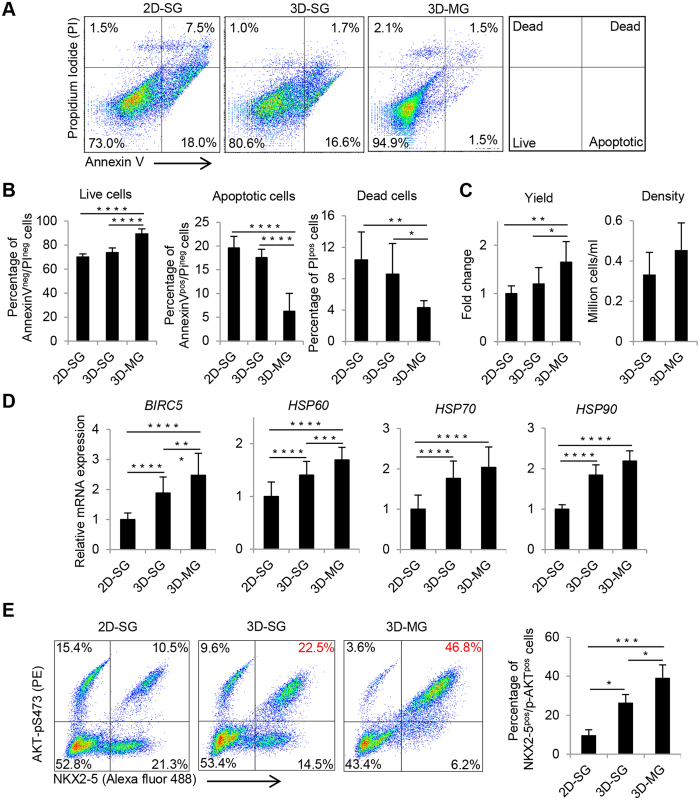
Simulated microgravity and 3D culture increase cell viability of cardiac progenitors. (**A**) Representative flow cytometry plots of viability of the cells at day 8 and assessed by Annexin V and PI. The nature of the cells in each quadrant is noted in the far-right diagram. (**B**) Summary of the viability, apoptotic and dead cells at day 8. Data are presented as mean ± SD of 8 biological replicates. (**C**) Summary of the cell yield and density. Yield was calculated based on the number of viable cells at day 8 generated from each input undifferentiated stem cell. Data are presented as mean ± SD of 8 biological replicates. (**D**) Relative expression levels of genes associated with pro-survival in cells at differentiation day 8 according to qRT-PCR. Data are presented as mean ± SD of 8 biological replicates x 3 reactions/sample. (**E**) Representative flow cytometry analysis and summary of cells at differentiation day 8 that were co-stained with p-AKT and NKX2-5. Data are presented as mean ± SD of 3 biological replicates *P < 0.05; **P < 0.01; ***P < 0.001.

To examine if suspension culture and exposure to simulated microgravity could improve cell survival, we assessed cell survival at day 8 before and after dissociation of cultures. Live/Dead staining of intact cultures showed that 3D-MG contained less dead cells than 3D-SG and 2D-SG (Fig. S3C). To quantify the difference, we stained the dissociated cells using propidium iodide (PI), which stains dead cells, and FITC-labeled Annexin V, which stains apoptotic cells. In 3D-MG, ~90% of the cells were viable, whereas in 2D-SG and 3D-SG, ~70% and ~74% of the cells were viable, respectively (Fig. 7A,B); the proportions of live cells in 3D-MG were higher than 3D-SG and 2D-SG (P < 0.0001) while the proportions of apoptotic or dead cells were significantly lower in 3D-MG than 3D-SG and 2D-SG (P < 0.0001 to 0.05).

In addition, the cell yield at day 8 generated from each input hPSC was higher in 3D-MG than 3D-SG and 2D-SG (P < 0.05 and <0.01, respectively, Fig. 7C). Cell density was also higher in 3D-MG than 3D-SG (Fig. 7C).

We next examined the expression of prosurvival genes in day 8 cells (Fig. 7D). Expression of *BIRC5,* which encodes an anti-apoptotic protein^34^, was detected at higher level in 3D cultures than 2D culture (P < 0.0001) and in 3D-MG than 3D-SG (P < 0.001). Similarly, expression of genes encoding heat shock proteins *HSP60*, *HSP70* and *HSP90* was detected at higher level in 3D cultures than 2D culture (P < 0.0001), and the level of *HSP60* expression was higher in 3D-MG than 3D-SG (P < 0.001).

We then examined day 8 cardiac progenitor cells for their expression of p-AKT (phosphorylated AKT-pS473), which plays a critical role in regulating cell survival^35^,^36^ (Fig. 7E and 7F). By flow cytometry, ~10%, ~27% and ~39% of NKX2-5^pos^/p-AKT ^pos^ cells were detected in 2D-SG, 3D-SD and 3D-MG, respectively; the proportions of NKX2-5^pos^/p-AKT ^pos^ cells in 3D-MG were higher than 3D-SG and 2D-SG (P < 0.05 and <0.001, respectively), and also higher in 3D-MG than 3D-SG (P < 0.05) (Fig. 7E).

To further understand how simulated microgravity affects cardiac progenitors during early stage of cardiac differentiation, RNA-sequencing analysis was performed to compare global gene expression profiles of day 8 cells in 3D-MG vs. 3D-SG. Among differentially expressed genes, 53 of them were up-regulated and 75 were down-regulated. The top 30 up-regulated genes (Table S1) include *LEPR*, liptin receptor, which is involved in fatty acid metabolism required for mature CMs; *HCN4*, which is a marker for the first heart field cardiac progenitors and conduction systems^37^; *HAND2* which is essential for cardiac ventricle formation^38^; and *LIFR*, leukemia inhibitory factor receptor, which is involved in AKT signaling that is important for CM growth, survival and function^39^.

Together, these data demonstrate increased cell viability and increased expression of genes associated with growth, development, and pro-survival in 3D-MG than 3D-SG at the progenitor cell stage.

## Discussion

The effect of simulated microgravity on cells is known to vary depending on factors including cell type, specific bioreactor used and duration of exposure to simulated microgravity^40^,^41^,^42^. Our study suggests that a short-term exposure of cardiac progenitors to simulated microgravity in an RPM may be leveraged to improve the expansion and viability of cardiac progenitors and subsequent generation of CMs. In combination with the engineering of microscale 3D cardiac progenitors, the exposure of the cells to simulated microgravity significantly increased cell viability (up to 90%), purity (up to 99%) and yield (up to 8-fold higher) of hPSC-CMs compared with the conventional 2D culture under standard gravity. In addition, CM yield was 1.5 to 4-fold higher in 3D-MG than 3D-SG.

Although 2D cardiac differentiation protocols using monolayer methods are available to produce CMs at high purity^2^,^13^, differentiation efficiency among different cell lines or batches varies. Methods such as depletion of glucose from medium^43^ have been developed to enrich CMs at late stages of differentiation when the differentiation efficiency is low. The culture system we have established using simulated microgravity and 3D culture provides a novel way to increase the induction, proliferation and survival of cardiac progenitors, thus further improving CM cell numbers (yield) and purity, an outcome that is desirable for the application of hPSC-CMs in regenerative medicine. Furthermore, this culture system could help identify molecular mechanisms associated with improved the induction of cardiac progenitors and develop alternative strategies under standard gravity to improve CM differentiation.

The high purity, viability and yield of hPSC-CMs at the late stage of differentiation are also likely contributed by the increased proliferation and viability of cardiac progenitors at the early stage of differentiation. Compared with the 2D culture, the early stage 3D cultures contained more cells with features of proliferation (measured by the incorporation of EdU and expression of Ki-67) and higher expression levels of genes related to cell proliferation and cell cycle. Furthermore, the 3D culture and simulated microgravity improved the viability of cardiac progenitors and up-regulated pro-survival genes that encode heat shock proteins and anti-apoptotic protein BIRC5 (also known as survivin). The HSP70/90 heat shock proteins are known to be involved in the regulation of cell cycle progression and cell proliferation^44^, and increased expression of *BIRC5* should result in less apoptosis that might be induced by microgravity stress. In other studies, up-regulation of heat shock proteins was also observed in rat hypothalamus tissues^45^, the Jurkat cells^46^ and myelomonocytic U937 cells^47^ as a result of exposure to microgravity, and *BIRC5* was up-regulated in thymus after mice returned from a spaceflight^48^. In addition, a study on neonatal and adult cardiovascular progenitors reported that simulated microgravity could affect cell differently depending on age^42^. Among many differential responses, final number of cells and expression of DNA repair genes were increased in neonatal but not adult cardiac progenitors upon exposure to simulated microgravity^42^. These studies suggest that simulated microgravity can improve cell proliferation and survival in a cell type dependent manner.

Given the high viability of CMs and higher expression levels of genes associated with proliferation and pro-survival including *HSPs* and *BIRC5*, the cardiac sphere preparation generated under simulated microgravity may be desirable for regenerative medicine over CMs generated from the 2D culture. Up-regulation of heat shock proteins through a brief treatment of heat shock has been shown to increase cell survival in both *in vitro* and *in vivo* studies with hPSC-CMs^49^. The transcriptional mechanisms and the biological meaning of the higher expression of *HSPs* and *BIRC5* in 3D-MG hPSC-CMs remain to be elucidated. These 3D-MG cells with high levels of *HSPs* and *BIRC5* might be more resistant to cell death and improve graft survival *in vivo*. Further understanding the mechanisms and pathways involved in the regulation of cell survival by simulated microgravity could facilitate the identification of alternative methods under standard gravity for efficient production of cardiac progenitors and CMs with enhanced potential for graft survival. Further understanding the mechanisms and pathways involved in the regulation of cell survival by simulated microgravity could facilitate the identification of alternative methods under standard gravity for efficient production of cardiac progenitors and CMs with enhanced potential for graft survival.

We have not examined the effect of longer exposure to simulated microgravity as we intended to target cardiac progenitors at the early stage of the differentiation rather than CMs at the late stage. Our study was designed to expand cardiac progenitors at differentiation days 5 or 6 under a brief exposure to simulated microgravity (3 days) and then transfer them to standard gravity for further differentiation into CMs, because a long-term exposure of microgravity could have a negative effect on stem cell differentiation. In fact, a long-term exposure of undifferentiated mouse ESCs to space microgravity for 15 days inhibited their differentiation^50^. Thus, the duration of microgravity treatment is an important factor to be considered, although the discrepancy in experimental outcomes between this mouse ESC study^50^ and our study may also be due to differences in cell types used (mouse ESCs vs. hPSCs), stages of the cells exposed to microgravity (undifferentiated cells vs. cardiac progenitors), types of microgravity (space microgravity vs. simulated microgravity) and/or environmental factors (with and without radiation and other factors in space and during transportation). In addition, longer exposure to simulated microgravity could also adversely affect the properties of CMs. Prolonged space flight can induce health problems including muscle atrophy and cardiovascular disorders^51^. CMs produced in our study, however, had expected molecular and functional features. 3D-MG increased the number of cells with the well-organized sarcomeric structure and increased the expression of genes encoding cardiac contractile proteins and calcium handing proteins. 3D-MG CMs also had appropriate pharmacological responses and improved Ca^2+^ handling properties. The action potential profiles of 3D-MG CMs were similar to those of 3D-SG CMs for all parameters except faster dV/dtmax in 3D-SG CMs. The underlying mechanisms of these observations still require to be further investigated.

In conclusion, we demonstrate that suspension culture and expansion of 3D engineered progenitor cardiac spheres under simulated microgravity result in efficient generation of highly enriched hPSC-CMs. This culture system increases proliferation and viability of cardiac progenitors, which could improve the application of these cells in regenerative medicine.

## Methods

Detailed methods are available in the Online Supplementary Information. In brief, IMR90 iPSCs^52^ and hESCs (H7 and H9)^53^ were used to generate cardiac progenitors by growth factors^2^,^54^ or small molecules^13^. Progenitor cardiac spheres were generated by forced aggregation in AggreWells^28^ and subjected to simulated microgravity using an RPM^55^,^56^. Characterization was performed to compare cells under standard gravity and simulated microgravity using various methods^28^,^57^,^58^ including microscopy, immuonocytochemical analysis, flow cytometry, qRT-PCR, RNA-seq, MEA recordings, calcium imaging, and patch-clamp.

## Additional Information

**Accession code**: The RNA-seq data reported in this paper are available on the GEO database with accession number GSE84582.

**How to cite this article**: Jha, R. *et al*. Simulated Microgravity and 3D Culture Enhance Induction, Viability, Proliferation and Differentiation of Cardiac Progenitors from Human Pluripotent Stem Cells. *Sci. Rep.* **6**, 30956; doi: 10.1038/srep30956 (2016).

## Supplementary Material

Supplementary Information

Supplementary movie S1

Supplementary movie S2

Supplementary movie S3
