# Supplementary material for: Simulated Microgravity and 3D Culture Enhance Induction, Viability, Proliferation and Differentiation of Cardiac Progenitors from Human Pluripotent Stem Cells
Source: Sci Rep. 2016 Aug 5;6:30956. doi: 10.1038/srep30956 (PMC4974658; doi:10.1038/srep30956)
Supplement: Supplementary Information [file srep30956-s1.pdf]

Supplementary Information

Simulated Microgravity and 3D Culture Enhance Induction, Viability, Proliferation and Differentiation of Cardiac Progenitors from Human Pluripotent Stem Cells

Rajneesh Jha, Qingling Wu, Monalisa Singh, Marcela K. Preininger, Pengcheng Han, Gouliang Ding, Hee Cheol Cho, Hanjoong Jo, Kevin O. Maher, Mary B. Wagner, Chunhui Xu

Supplemental Results

Effect of the duration of simulated microgravity exposure on CM differentiation

We determined the optimal duration of microgravity exposure to produce robust CM differentiation with high viability and purity. Day 4 IMR90 iPSCs differentiated cells were dissociated and seeded at 1500 cells/microwell to form progenitor cardiac spheres. At day 5, approximately 2400 progenitor cardiac spheres in 10 ml of RPMI-B27 medium were loaded into each Opticell disk and exposed to MG during days 5-6 (3D-MG 1 day), days 5-8 (3D-MG 3 days) and days 5-10 (3D-MG 5 days) and then transferred to SG (Fig. S2). Parallel 2D-SG and 3D-SG cultures were maintained as controls.

At day 20, these cultures were analyzed for cell viability and CM purity by flow cytometry (Fig. S2D). Both 3D-SG and 3D-MG with all durations of MG exposure significantly enhanced cell viability as compared to the 2D-SG culture ($P<0.001$ to 0.05). Among 3D cardiac sphere cultures, only cells in the 3D-MG 3 days culture showed significantly higher viability as compared to 3D-SG ($P<0.05$) (Fig. S2E). As detected by α-actinin staining, highly enriched CMs (>80%) were generated in 3D cultures (Fig. S2D). Similar to the viability results, the purity of 3D-SG and 3D-MG cardiac spheres was significantly higher than that of 2D-SG ($P<0.0001$ to 0.05). Among 3D cells, cells exposed to MG for 3 and 5 days had significantly higher purity than cells exposed to MG for 1 day ($P<0.05$) (Fig. S2E). This suggests that a short exposure to MG for 1 day may be less effective to enrich CMs.

The yield of CMs per input of iPSC in 3D cultures was significantly (2-4 fold) higher than that of 2D-SG culture ($P<0.0001$ to 0.05). Among 3D conditions, 3D-SG and 3D cells exposed to MG for 1 day produced similar level of CMs, again suggesting that a short exposure to MG for 1

day may not be sufficient for robust CM differentiation. However, 3D cells exposed to MG for 3 and 5 days significantly enhanced the yield of CMs when compared to 3D-SG ($P<0.01$ and 0.05 respectively). In addition, we observed a significant increase in the yield of 3D culture exposed to MG for 3 days but not 5 days in comparison with the 3D culture exposed to MG for 1 day ($P<0.05$) (Fig. S2E).

Overall, in terms of viability, purity and yield, a 3 day exposure of 3D cells to MG was the optimal condition for robust generation and enrichment of CMs.

Supplemental Materials and Methods

Cell culture and CM differentiation. Human iPSCs derived from IMR90 fibroblasts (WiCell Research Institute, Inc.)[1] and hESCs (H7 and H9, WiCell Research Institute, Inc.)[2] were maintained in mouse embryonic fibroblast-conditioned medium supplemented with 8 ng/ml basic fibroblast growth factor[3]. After cultured for 5 to 7 days until stem cell colonies occupied ~80% of the surface area, cells were dissociated with Versene (EDTA) and reseeded for CM differentiation using either growth factors (activin A and BMP4, R&D Systems)[4] or small molecules (CHIR99021 and Wnt-C59, SelleckChem)[5]. For growth factor-guided differentiation, cells were seeded on Matrigel-coated plates and cultured until compact colonies covered the wells. At the day of induction (day 0), the medium was replaced with RPMI 1640 medium supplemented with 2% B27 without insulin and 100 ng/ml activin A. After 24 h (at day 1), activin A was replaced with RPMI 1640 medium supplemented with 2% B27 without insulin and 10 ng/ml bone morphogenetic protein 4 (BMP4). Then, induced cultures were cultured for additional 4 days without any medium change. At day 5, the BMP4-containing medium was replaced with RPMI 1640 medium supplemented with 2% B27 with insulin. For differentiation using small molecules[5], dissociated single cells were seeded on Matrigel-coated plates in E8 medium and cultured till reaches ~85% confluence. On day 0, cultures were treated with CHIR99021 (6 µM) in RPMI 1640 supplemented with 2% B27 without insulin for 48 h followed by medium change. At day 3, cultures were treated with Wnt-C59 (2 µM) in RPMI 1640 medium supplemented with 2% B27 without insulin and incubated for another 2 days. At day 5, medium was replaced with RPMI 1640 medium supplemented with 2% B27 with insulin and changed on alternate days afterwards. Cells were observed under a microscope daily for beating cells, which typically appeared after day 8-10, and were harvested at day 20 to determine CM yield and purity.

To examine the effect of dissociation and replating cardiac progenitors in 2D culture on CM differentiation, day 4 cells were dissociated and re-seeded at low ($1.1x10^5$ cells/cm^2), medium ($2.1x10^5$ cells/cm^2) and high ($4.2x10^5$ cells/cm^2) densities on Matrigel-coated plates in 2D culture. At day 20, cells were harvested and analyzed for CM differentiation by flow cytometry analysis. Parallel intact cultures were used as a control.

Formation of progenitor cardiac spheres. At differentiation day 4 or 5, cells were dissociated with 0.25% trypsin/EDTA and seeded into AggreWell 400 plates (Stem Cell Technologies) to aggregate into cardiac spheres. Before cell seeding, plates with 0.5 ml/well of RPMI+B27 medium were centrifuged at 1000 g to release trapped bubbles in microwells. The density of 1500 cells/microwell was selected based on initial optimization by adding cells at 500, 1500 and 2500 cells/microwell (Fig. S1D). To prevent cell death, medium was supplemented with 10 µM of Rock inhibitor Y-27632. Plates were centrifuged at 100 g to distribute the cells and then placed in an incubator. After 24 h spheres were transferred to suspension culture.

Culture of 3D progenitor cardiac spheres in suspension under standard gravity and simulated microgravity. At day 5 or 6, ~2400 progenitor cardiac spheres were injected through a syringe and a wide-bore needle into a 100 mm low attachment petri dish for 3D-SG or gas-permeable OptiCell disk (Thermo Scientific) for 3D-MG. The OptiCell disk containing spheres was completely filled with medium (up to 14 ml) to devoid of air bubbles and sealed. The spheres in the petri dish were cultured under standard gravity and the spheres in the OptiCell disk were cultured under simulated microgravity using a random positioning machine (RPM) [6, 7]. The OptiCell disk was fixed close to the center of the platform in the RPM which was positioned in the same incubator as the 3D control cultures under standard gravity. A parallel 2D culture under standard gravity was also maintained as a control. Medium was changed every 2 days and spheres exposed to simulated microgravity for 3 days and then transferred to standard gravity.

The RPM is a 3D clinostat that can simulate microgravity by altering the position of the platform through two frames, an inner frame and an outer frame which can continuously rotate in a random mode, where rotation speeds and directions are randomized using a software developed by the Dutch Space Agency (version 1.3.5, Leiden, the Netherlands). This continuous movement of the gravity vectors averages the vector to zero over time based on a method of "gravity-vector-

averaging", allowing cells in the OptiCell disks on the platform to be exposed to simulated microgravity[7, 8].

Flow Cytometry. Flow cytometry analysis was performed as described previously[9]. Cells were harvested at appropriate time points by incubating with 0.25% trypsin-EDTA at 37ºC for 10 min and subsequently neutralized by 10% FBS in DMEM. For the dissociation of differentiation cultures, 2D cells were triturated for 5-10 times and 3D cardiac spheres were triturated about 20-25 times because they were harder to be dissociated and required more trituration. Cells were counted, aliquoted at 5×10^5 cells/ test, and used for staining target proteins and for corresponding isotype controls and compensation controls. Then cells were washed once with PBS (5 ml/tube) and suspended in 0.5 ml staining buffer (PBS containing 2% heat-inactivated FBS) containing ethidium monazide (EMA, a dye for the detection of dead cells) at 1 µg/ml and incubated in dark on ice for 15 min. Further, cells were pelleted, resuspended in 1 ml PBS and exposed to bright light for 10 min by placing tubes horizontally on ice bed. Cells were washed once with 2 ml PBS and fixed in 2% paraformaldehyde (PFA) at room temperature for 15 min by adding 4% PFA (0.5 ml) in equal volumes of cell suspension. For surface staining, cells were blocked with 20% normal goat serum in staining buffer for 30 min and then incubated with conjugated antibody for 30 min on ice, washed two times and stained with Live/Dead fixable dead cell staining kits (Life Technologies) and further analyzed by flow cytometry. For intracellular staining, cells were washed again, resuspended in 100 µl of PBS, permeabilized by adding 900 µl of ice cold absolute methanol, and then incubated on ice for 30 min. Cells were washed once and blocked with blocking solution (20% normal goat serum [Invitrogen] in staining buffer) at room temperature for 30 min. After blocking, cells were washed and incubated for 20 min at room temperature with primary antibody or isotype control in 100 µl of BS. Cells were washed 2 times and incubated for 15 min at room temperature in dark with the corresponding secondary antibodies. Cells were then again washed 3 times and resuspended in 200 µl of staining buffer. For phosphorylated-AKT staining, cells were fixed in Fix Buffer I (BD Biosciences) for 10 min at 37ºC then permeabilize with BD Perm Buffer III on ice for 30 min and further staining procedure was followed.

For multi-color flow cytometry analysis of surface markers for cardiac progenitors (CD13, KDR and PDGRRα) and for CMs (SIRPA), single cell suspension ($1\text{x}10^6$ cells/test) were blocked using 20% normal goat serum and then incubated with a mixture of antibodies in 100 µl on ice for

30 min. The cells were washed two times with 4% FBS in D-PBS and subsequently stained with Fixable Live/Dead Near-IR (Life Technologies) for 30 min on ice following manufacturer's instruction. Cells were fixed in 2% PFA for 30 min at room temperature, washed and kept at 4°C till flow cytometric acquisition.

For flow cytometry analysis of intracellular markers for CMs (cTnT and α-actinin) or cardiac progenitors (ISL1), cells were stained with Fixable Live/Dead Near-IR and fixed in 2% PFA, subsequently cells were permeabilized by incubating with absolute methanol on ice for 30 min and then blocked using 20% normal goat serum. Further, cells were stained with either conjugated antibodies or sequentially stained with primary and secondary antibodies.

Stained cells were analyzed by BD FACS Canto II or FACS Aria II by adjusting voltage and color compensation using appropriate excitation and detection channels. Forward versus side scatter quadrants were defined and at least 10,000 live cells were acquired. Finally, dot plots were generated upon data analysis using FlowJo software to display the percentage of cell populations. All antibodies are listed in Table S2.

Of note, the majority of α-actinin^{pos} cells in our CM differentiation cultures were also positive for CM-specific markers. Almost all α-actinin^{pos} cells were positive for a CM-specific transcription factor, NKX2-5, as detected by immunostaining (Fig. S8A). Similarly, ~95-97% of the α-actinin^{pos} cells were positive for another CM-specific marker, cTnT (cardiac troponin T), as examined by flow cytometry analysis (Fig. S8B). These results were consistent with previous studies showing that that almost all α-actinin^{pos} cells were co-stained with NKX2-5[10] and that the percentage of α-actinin^{pos} cells was similar to the percentage of cTnT^{pos} in CM differentiation cultures derived from hESCs[11]. Thus, flow cytometry of α-actinin was used to identify CMs in differentiation cultures in this study.

We selected NKX2-5 as a marker to monitor proliferation of cardiac cells since NKX2-5 is a transcription factor that is associated with precardiac mesoderm and persists in the heart during development. As shown in our previous study[10], the expression of NKX2-5 was detectable as early as differentiation day 6 and increased overtime. At differentiation day 8, about a half of NKX2-5^{pos} cells were also positive for α-actinin, a CM-associated sarcomeric protein, and by day 23, almost all NKX2-5^{pos} cells were positive for α-actinin[10].

Immunocytochemical analysis

Differentiation cultures were dissociated and re-plated on a Matrigel-coated 96 well culture plate at a density of 2-5x10^4 cells/well and cultured for two days before fixation. On the day of immunostaining, cells were washed with cold PBS, fixed in 2% PFA at room temperature for 15 min, and permeabilized in cold ethanol for 2 min at room temperature. Cells were washed and blocked with 3% BSA or 5% normal goat serum in PBS at room temperature for 1-2 h and incubated with the primary antibodies specified in Table S2 in 3% BSA or 1% normal goat serum overnight at 4°C. After incubation with the primary antibody, cells were washed thrice with PBS for 5 min each with gentle agitation to get rid of the unbound primary antibody. Cells were then incubated with the corresponding conjugated secondary antibody at room temperature for ~45 min, in the dark, then washed thrice with PBS and counterstained with Vectorshield mounting medium containing DAPI (Vector Laboratories, #H-1200). Imaging was performed using the inverted microscope (Axio Vert.A1).

qRT-PCR

Total RNA was extracted from differentiation cells at day 8 or at day 20 according to the manufacturer's recommendation using Aurum total RNA mini kit (Bio-Rad). Briefly, ~2x10^6 cells were rinsed once with 1 ml PBS and replaced with 350 µl lysis solution supplemented with 1% β-mercaptoethanol, then pipeted up and down several times to lyse the cells thoroughly. Lysed cells were collected in RNAse and DNAse–free 1.5 ml tubes and kept at -80°C until RNA isolation. Individual RNA sample (1 µg) was reverse transcribed by adding 100 U of Superscript III enzyme and random primers in 20 µl reaction mixture containing Vilo reaction buffer as per manufacturer's instruction in SuperScript VILO™ cDNA Synthesis Kit (Life Technologies). Reaction mixture was then incubated at 3 different temperature cycles: 25°C for 10 min, 42°C for 2 h and 25°C for 5 min in Bio-Rad C1000 touch thermal cycler. One reaction without superscript III (minus RT) was also used as negative control. Further, reaction mixture was diluted 10 times to 200 µl and 2 µl cDNA was used for real-time PCR. Human specific PCR primers (Table S3) for different genes were retrieved from open access websites (http://primerdepot.nci.nih.gov/ or http://pga.mgh.harvard.edu/primerbank/) and all primers were synthesized by IDT at 25 nmoles scale. Real-time PCR reaction was performed in 20 µl mixture containing 2x iTaq SyBr green master mix, 2 µl of cDNA mix, and 200 nM of forward and reverse primers. Reaction cycle was initial denaturation step at 95°C for 10 min, 40 cycles of two-step PCR with 15 sec of denaturation

at 95°C followed by 1 min of annealing/extension at 60°C. A melt curve was generated for all reactions to check for product integrity and primer-dimer formation as default settings in Applied Biosystems 7500 real-time PCR systems. mRNA levels for each sample were normalized to GAPDH mRNA levels and then expressed as a relative fold changes as compared with mRNA levels in 2D-SG control.

Calcium imaging

Differentiation cultures at day 20 were dissociated with 0.25% trypsin-EDTA and seeded onto sterile, Matrigel (1:30 dilution)-coated 25x25x1 mm glass coverslips and cultured for 10-30 days. Live cell imaging of intracellular calcium transients was performed to assess functional characteristics of CMs. The cells on coverslips were incubated with 5 μM of the calcium dye Fluo-4 AM (Life Technologies) for 15 min at 37°C, then transferred to a temperature-controlled microscope chamber and perfused at 37°C with modified Tyrode's solution (140 mM NaCl, 4 mM KCl, 2 mM $CaCl_2$, 1 mM $MgCl_2$, 10 mM HEPES, and 5 mM glucose, pH adjusted to 7.4 with NaOH)[12]. Recordings of intracellular calcium fluorescence were acquired when the cells were paced at 1 Hz at 40x magnification using an inverted laser confocal scanning microscope Olympus FV1000 equipped with FluoView software (Olympus). The recordings were exported and analyzed with ClampFit 10.0 software (Molecular Devices) as described[13].

Electrophysiological measurements of action potentials

Action potentials of single beating cells were recorded in a current-clamp mode using an Axon 200B amplifier at 37°C. Patch glass pipettes were filled with an intracellular solution containing (in mM) 9 KCl, 130 K- glutamate, 8 NaCl, 0.5 MgCl2, 5 Mg-ATP, 10 EGTA, and 10 HEPES to the final resistance of 3-5 MΩ. The pH was adjusted to 7.3 with KOH. The perfusion bath solution was normal Tyrode's containing (in mM) 140 NaCl, 5 KCl, 1 $CaCl_2$, 1 $MgCl_2$, 10 glucose, and 10 HEPES. The pH was adjusted to 7.4 with NaOH.

Data were digitized at 10 kHz, low-pass filtered at 5 kHz, and analyzed with pClamp 9. Electrophysiological parameters measured included: N, cell number; dV/dt, max, maximum action potential upstroke velocity; MDP, maximum diastolic membrane potential; APA, action potential amplitude; APD50, action potential duration at 50% of repolarization; APD90, action potential duration at 90% of repolarization. The CMs were categorized as a nodal type if all of the following

three criteria were met[10]: a MDP ≥ −50 mV, an APA ≤ 90 mV, and APD90/APD50 ≥ 1.7. Following these criteria, the ‘nodal’ type myocytes exhibited a substantially slower dV/dt_{max}, which is one of the distinguishing features of the native nodal pacemaker cells’ action potential morphology. All other CMs were categorized as working-type CMs. We did not sub-classify the working-type CMs into ventricular and atrial CMs due to the immature nature of hPSC-CMs. Immature human (neonatal) atrial CMs have longer ADP50 and ADP30 than adult atrial CMs[14], thus it is difficult to accurately distinguish immature atrial CMs from immature ventricular CMs. N = 5-10 cells of each subtype from 3D-MG were compared with those from 3D-SG.

Microelectrode array recording

Electrophysiological properties and pharmacological responses of CMs were analyzed using a 64-channel Muse microelectrode array (MEA) data acquisition system (Axion Biosytems). MEA chambers were cleaned with absolute ethanol, dried and subsequently incubated for 1 h with fibronectin (50 µg/ml). CMs at differentiation day 20 generated from 2D-SG, 3D-SG and 3D-MG cultures were re-plated onto fibronectin-coated MEA chambers. Cells were maintained in RPMI-B27 medium for additional 2 days to recover beating before the recording. Using this system, extracellular potentials of CMs were detectable from electrodes on an MEA chamber. To examine the effect of pharmacological drugs at different concentrations, cells were treated with isoproterenol (1 µM), carbamylcholine (10 µM), phenylephrine hydrochloride (100 µM), and nifedipine (1 µM and 10 µM). Extracellular recordings were performed for 1 min at the baseline prior to the application of drugs. Further, cells were incubated with drugs for 5 min at 35°C and pharmacological responses were then recorded for 1 min. Beating frequency was analyzed with the AxIS software and Excel. Field potential duration (FPD) was analyzed with the Clampfit 10.4 software and Excel, and normalized mean corrected FPD (cFPD) was generated by normalizing FPD with the beating rate[15].

Cell viability staining. Live and dead staining was performed before and after dissociation of 2D-SG, 3D-SG and 3D-MG cultures using LIVE/DEAD Viability/Cytotoxicity Kit and FITC Annexin V and propidium iodide (PI) kit (Life Technologies), respectively. For the staining of intact cultures, cultures at day 8 and day 20 were washed with phenol red-free RPMI 1640 medium and incubated for 30 min in the same medium containing ethidium homodimer (1 µM) and calcein

(0.5 μM) for the detection of dead and live cells, respectively. Cultures were then washed twice with PBS, resuspended in the phenol red-free RPMI 1640 medium and imaged using a microscopy. Ethidium homodimer enters through the dead and damaged cells and produces bright red fluorescence (ex/em ~495 nm/~635 nm) upon binding to nucleic acids. The live cells were detected by the presence of ubiquitous calcein with strong green fluorescence (ex/em ~495 nm/~515 nm).

For Annexin V/PI staining, cultures at day 8 were dissociated using 0.25% trypsin-EDTA and analyzed for cell viability using flow cytometry. Single cells ($1x10^6$ cells/test) were washed with PBS and co-stained using PI, which stains dead cells, and FITC-conjugated Annexin V, which stains apoptotic cells because it has high affinity for phosphatidylserine, a phospholipid that is normally found inside the cell membrane but is transferred to the outer layer of the cell membrane when cells undergo apoptosis [16]. Cells were resuspended in 5 μl of FITC-conjugated Annexin V in 100 μl of Annexin labeling solution containing 10 mM HEPES (pH 7.4), 140 mM NaCl, 5 mM $CaCl_2$, and 1 μl of PI (100 μg/ml) and incubated at room temperature for 15 min. After the incubation period, 400 μl of 1X Annexin-binding buffer was added to the cells and mixed gently, and samples were kept on ice. At least 10,000 events were analyzed immediately by flow cytometry using the fluorescence emission at 530 nm (FL1) and >575 nm (FL3). The populations were separated into 3 groups: (1) live cells showing only a low level of fluorescence (Annexin $V^{neg}PI^{neg}$), (2) apoptotic cells showing green fluorescence (Annexin $V^{pos}PI^{neg}$), and (3) dead cells showing both red and green fluorescence (Annexin $V^{pos}PI^{pos}$).

Proliferation assay. Cardiac progenitors at differentiation day 8 were dissociated and re-seeded on a Matrigel-coated 96-well plate at a density of 2-5 x 10^4 cells/well for immunocytochemical analysis. The 5-ethynyl-2´-deoxyuridine (EdU) staining was done using Click-iT Plus EdU Imaging Kit (Life Technologies, #C10639). One day after cell seeding for immunocytochemical analysis and intact cultures for flow cytometry (day 9), cells were washed twice with 3% bovine serum albumin (BSA) in PBS and replaced with 100 μl or 500 μl of the fresh medium per well. After incubation at 37°C for about 2 h, cells were treated with 100 μl or 500 μl of 2X working solution in RPMI B27 medium containing EdU (final concentration 10 μM) and incubated overnight at 37°C. The EdU, a nucleoside analogue of thymidine, can be incorporated selectively during active DNA synthesis, hence the proliferative cells will incorporate EdU in their DNA [17].

Next day (day 10), cells were washed twice for immunocytochemical analysis with 3% BSA in PBS and fixed with 4% PFA for 15 min and permeabilized in cold ethanol for 2 min at room temperature. For flow cytometry, cells were washed twice and fixed with 2% PFA for 15 min at room temperature and permeabilized with ice-cold methanol for 30 min on ice in dark. Further, blocking was performed using 20% normal goat serum for 30 min at room temperature. Cells were washed and incubated with the EdU Click IT cocktail containing Click-iT reaction buffer, $CuSO_4$, Alexa Fluor 594 Azide, and reaction buffer additive according to the manufacturer's guidelines for 30 min in the dark at room temperature. Further, EdU-treated cells were incubated with NKX2-5 primary antibody and a separate staining was also performed for the co-staining of NKX2-5 with Ki-67 or IAK1 in PBS containing 3% BSA for 1 h at room temperature in dark and then washed thrice with PBS for 5 min each with gentle agitation to get rid of the unbound primary antibody. Cells were then incubated with the corresponding conjugated secondary antibody (Anti-rabbit IgG-Alexa 594 and Anti-mouse IgG-Alexa 488) in the dark for about 30-45 min. For immunocytochemical analysis, cells were washed thoroughly and counterstained with Vectorshield mounting medium containing DAPI (vector laboratories, #H-1200) and imaging was performed using the inverted microscope (Zwiss Axio Vert.A1). For flow cytometry, cells were washed 3 times and acquired on BD LSR II machine using appropriate emission and excitation channels and dot plots were generated.

RNA-Seq library preparation and sequencing

Libraries were prepared using the Illumina TruSeq RNA kit (Illumina Inc. San Diego, CA, USA) as per manufacturer's instructions. Briefly, 1000 ng of total RNA samples from 3 independently derived cardiac progenitors at day 8 were used for library preparation. The TruSeq method (Low-Throughput protocol) employs two rounds of poly-A based mRNA enrichment using oligo-dT magnetic beads followed by mRNA fragmentation using cations at high temperature. First and second strand cDNA synthesis was performed followed by end repair of the blunt cDNA ends. One single "A" base was added at the 3' end of the cDNA followed by ligation of barcoded adapter unique to each sample. The adapter-ligated libraries were then enriched using PCR amplification. The amplified library was validated using a High Sensitivity DNA chip on the Agilent Bioanalyzer. The libraries were further quantified on Qubit 2.0 Fluorometer (Life Technologies, Grand Island, NY, USA) using the High Sensitivity dsDNA assay. The libraries were normalized and 8 samples

were multiplexed in each lane of the flowcell. PhiX was used as an internal control on each lane to monitor the error statistics. Cluster generation was performed on the V3 flowcell on the Illumina cBot. The clustered flowcell was then sequenced on the Illumina HiSeq1000 system employing a single-end 101 cycles run and reads were demultiplexed using Illumina CASAVA version 1.8.2. Reads were mapped to the Human genome reference (hg19) using the STAR Aligner version 2.4.0g1. Transcript quantitation was done with htseq-count version 0.6.1pl using the hg19 RefSeq annotation downloaded from the UCSC Genome Browser. Further, normalized expression table was generated and significantly (adjusted $P < 0.1$) altered genes were identified using statistical software package DESeq2 version 1.6.3.

Statistical analysis

For the statistical analysis, data were transformed using formula Y'=arcsine(SQRT(Y)) to fit into the assumption of parametric statistical test[18]. Further, histogram and density plot prior to and after transformation of data were performed to check if residuals were similar or not. Since the residuals were similar, original data were shown as bar graphs and transformed data were analyzed by one-way ANOVA with post Turkey's multiple comparision test using GraphPad Prism 6 statistical package software. For MEA recordings, three separate random intercept models were performed and the data were considered clustered as same subject measured multiple times in the random intercept model and fit using an unstructured co-variance matrix. A post-hoc turkey-kramer multiple comparison procedure was used to make pair-wise comparisons between the three groups. Both raw and studentized residuals were visually assessed for normality using histogram and QQ-plots. For calcium imaging analysis, Mann-Whitney U test was performed using JMP statistical software package. All data were expressed as mean ± SD and P values <0.05 were considered as significant.

Supplemental References

1. Yu, J. et al. Induced pluripotent stem cell lines derived from human somatic cells. *Science* **318**, 1917-1920 (2007).
2. Thomson, J.A. et al. Embryonic stem cell lines derived from human blastocysts. *Science* **282**, 1145-1147 (1998).

3. Xu, C. et al. Feeder-free growth of undifferentiated human embryonic stem cells. *Nature Biotech.* **19**, 971-974 (2001).
4. Nguyen, D.C. et al. Microscale generation of cardiospheres promotes robust enrichment of cardiomyocytes derived from human pluripotent stem cells. *Stem Cell Reports* **3**, 260-268 (2014).
5. Burridge, P.W. et al. Chemically defined generation of human cardiomyocytes. *Nat Methods* **11**, 855-860 (2014).
6. Pardo, S.J. et al. Simulated microgravity using the Random Positioning Machine inhibits differentiation and alters gene expression profiles of 2T3 preosteoblasts. *Am J Physiol Cell Physiol* **288**, C1211-1221 (2005).
7. Patel, M.J. et al. Identification of mechanosensitive genes in osteoblasts by comparative microarray studies using the rotating wall vessel and the random positioning machine. *J Cell Biochem* **101**, 587-599 (2007).
8. van Loon, J.J. Some history and use of the random positioning machine, RPM, in gravity related research. *Adv Space Res* **39**, 1161-1165 (2007).
9. Jha, R., Xu, R.H. & Xu, C. Efficient differentiation of cardiomyocytes from human pluripotent stem cells with growth factors. *Methods Mol Biol* **1299**, 115-131 (2015).
10. Xu, C. et al. Efficient generation and cryopreservation of cardiomyocytes derived from human embryonic stem cells. *Regen Med* **6**, 53-66 (2011).
11. Zhu, W.Z. et al. Neuregulin/ErbB signaling regulates cardiac subtype specification in differentiating human embryonic stem cells. *Circ Res* **107**, 776-786 (2010).
12. Wagner, M.B., Wang, Y., Kumar, R., Tipparaju, S.M. & Joyner, R.W. Calcium transients in infant human atrial myocytes. *Pediatr Res* **57**, 28-34 (2005).
13. Lee, Y.K. et al. Calcium homeostasis in human induced pluripotent stem cell-derived cardiomyocytes. *Stem Cell Rev* **7**, 976-986 (2011).
14. Wang, Y. et al. Differences in transient outward current properties between neonatal and adult human atrial myocytes. *J Mol Cell Cardiol* **35**, 1083-1092 (2003).
15. Navarrete, E.G. et al. Screening drug-induced arrhythmia events using human induced pluripotent stem cell-derived cardiomyocytes and low-impedance microelectrode arrays. *Circulation* **128**, S3-13 (2013).

16. Vermes, I., Haanen, C., Steffens-Nakken, H. & Reutelingsperger, C. A novel assay for apoptosis. Flow cytometric detection of phosphatidylserine expression on early apoptotic cells using fluorescein labelled Annexin V. *J Immunol Methods* **184**, 39-51 (1995).
17. Salic, A. & Mitchison, T.J. A chemical method for fast and sensitive detection of DNA synthesis in vivo. *Proc Natl Acad Sci U S A* **105**, 2415-2420 (2008).
18. McDonald, J.H. Data trasnformations. (Sparky House Publishing, 2014).

Table S1. List of Top 30 Genes Significantly Altered in 3D-MG as Compared with 3D-SG at Differentiation Day 8

Symbol	RefseqID	Gene name	log2 fold change	Adjusted P-value
Up-regulated				
CYP1B1	NC_000002.12	Cytochrome P450, Family 1, Subfamily B, Polypeptide 1	0.9809	0.0000
ARRDC3	NC_000005.10	Arrestin Domain Containing 3	0.8080	0.0000
CRLS1	NC_000020.11	Cardiolipin Synthase 1	0.5619	0.0000
NPR1	NC_000001.11	Atrial Natriuretic Peptide Receptor Type A	0.5577	0.0012
PKHD1L1	NC_000008.11	Polycystic Kidney And Hepatic Disease 1-Like 1	0.5357	0.0015
LEPR	NC_000001.11	Leptin Receptor	0.5277	0.0018
STAC	NC_000003.12	SH3 And Cysteine Rich Domain	0.5165	0.0013
H3F3B	NC_000017.11	H3 Histone, Family 3B (H3.3B)	0.5073	0.0000
FBXO25	NC_000008.11	F-Box Protein 25	0.4977	0.0088
ALX4	NC_000011.10	ALX Homeobox 4	0.4932	0.0175
ID4	NC_000006.12	Inhibitor Of DNA Binding 4, Dominant Negative Helix-Loop-Helix Protein	0.4532	0.0011
AKR1B10	NC_000007.14	Aldo-keto reductase family 1, member B10 (aldose reductase)	0.4518	0.0657
LRRN4	NC_000020.11	Leucine rich repeat neuronal 4	0.4419	0.0740
HCN4	NC_000015.10	Hyperpolarization activated cyclic nucleotide gated potassium channel 4	0.4392	0.0228
RHPN2	NC_000019.10	Rhophilin, Rho GTPase binding protein 2	0.4347	0.0015
CPNE7	NC_000016.10	Copine VII	0.4328	0.0776
LAMC1	NC_000001.11	Laminin, gamma 1	0.4235	0.0001
PCDH7	NC_000004.12	Protocadherin 7	0.4235	0.0005
ITGA3	NC_000017.11	Integrin, alpha 3	0.4217	0.0088
HPCAL4	NC_000001.11	Hippocalcin like 4	0.4104	0.0763
TSHZ2	NC_000020.11	Teashirt zinc finger homeobox 2	0.4094	0.0767
SLC7A7	NC_000014.9	Solute carrier family 7	0.4000	0.0961
COL21A1	NC_000006.12	Collagen, type XXI, alpha 1	0.3915	0.0503
DENND1B	NC_000001.11	DENN/MADD domain containing 1B	0.3879	0.0965
MFGE8	NC_000015.10	Milk fat globule-EGF factor 8 protein	0.3848	0.0063
BNC1	NC_000015.10	Basonuclin 1	0.3832	0.0111
HAND2	NC_000004.12	Heart and neural crest derivatives expressed 2	0.3795	0.0216
LIFR	NC_000005.10	Leukemia inhibitory factor receptor alpha	0.3777	0.0154
HES1	NC_000003.12	Hes family bHLH transcription factor 1	0.3776	0.0468

LSAMP	NC_000003.12	Limbic system-associated membrane protein	0.3519	0.0503
Down-regulated				
CDH11	NC_000016.10	Cadherin 11, type 2, OB-cadherin (osteoblast)	-0.4570	0.0001
ITGB6	NC_000002.12	Integrin, beta 6	-0.4572	0.0475
CDKN1A	NC_000006.12	Cyclin-dependent kinase inhibitor 1A (p21, Cip1)	-0.4592	0.0209
NKD1	NC_000016.10	Naked cuticle homolog 1	-0.4598	0.0175
CRABP2	NC_000001.11	Cellular retinoic acid binding protein 2	-0.4649	0.0180
FLJ16779	NC_000020.11	Uncharacterized LOC100192386	-0.4655	0.0083
SEMA6A	NC_000005.10	Sema domain, transmembrane domain (TM), and cytoplasmic domain, (semaphorin) 6A	-0.4658	0.0044
TNFRSF19	NC_000013.11	Tumor necrosis factor receptor superfamily, member 19	-0.4678	0.0186
ITGA2	NC_000005.10	Integrin, alpha 2 (CD49B, alpha 2 subunit of VLA-2 receptor)	-0.4688	0.0475
COL16A1	NC_000001.11	Collagen, type XVI, alpha 1	-0.4725	0.0364
WISP1	NC_000008.11	WNT1 inducible signaling pathway protein 1	-0.4725	0.0475
JAG1	NC_000020.11	Jagged 1	-0.4751	0.0292
BMP7	NC_000020.11	Bone morphogenetic protein 7	-0.4753	0.0412
EDNRB	NC_000013.11	Endothelin receptor type B	-0.4782	0.0404
KLK6	NC_000019.10	Kallikrein-related peptidase 6	-0.5000	0.0265
PDE1B	NC_000012.12	Phosphodiesterase 1B, calmodulin-dependent	-0.5040	0.0005
LYPD6B	NC_000002.12	LY6/PLAUR domain containing 6B	-0.5083	0.0216
NDNF	NC_000004.12	Neuron-derived neurotrophic factor	-0.5157	0.0004
THY1	NC_000011.10	Thy-1 cell surface antigen	-0.5389	0.0031
ELF3	NC_000001.11	E74-like factor 3 (ets domain transcription factor, epithelial-specific)	-0.5410	0.0034
RUNX1	NC_000021.9	Runt-related transcription factor 1	-0.5489	0.0093
SFRP1	NC_000008.11	Secreted frizzled-related protein 1	-0.5572	0.0000
APLNR	NC_000011.10	Apelin receptor	-0.5573	0.0013
GPRC5A	NC_000012.12	G protein-coupled receptor, class C, group 5, member A	-0.5588	0.0031
BHLHE40	NC_000003.12	Basic helix-loop-helix family, member e40	-0.5615	0.0068
KRT7	NC_000012.12	Keratin 7, type II	-0.5732	0.0033
NKAIN4	NC_000020.11	Na+/K+ transporting ATPase interacting 4	-0.5781	0.0017
P2RY6	NC_000011.10	Pyrimidinergic receptor P2Y, G-protein coupled, 6	-0.6239	0.0011
TGM2	NC_000020.11	Transglutaminase 2	-0.6841	0.0002
MMP9	NC_000020.11	Matrix metallopeptidase 9	-1.2768	0.0000

Note: RNA samples from 3 independently derived cardiac progenitors at day 8 were used.

Table S2. Antibodies

Type	Antibody target	Origin/isotype	Supplier	Catalog Number	Dilution or concentration	Application
Primary	α-actinin	Mouse IgG1	Sigma	A7811	4 ug/ml or 1:800	ICC, FCM
	IAK1	Mouse IgG1	BD Biosciences	610938	1:100	FCM
	Ki-67	Mouse IgG1	BD Biosciences	550609	5 ug/ml	ICC, FCM
	MESP1/2	Rabbit IgG	Aviva Systems Biology	ARP39374	1.25 ug/ml	FCM
	N-cadherin	Mouse IgG1	Sigma	C3865	40 or 80 µg/ml	ICC
	NKX2-5	Rabbit IgG	Santa Cruz Biotech	sc-14033	1:400	ICC, FCM
	Tra1-60	Mouse IgM	EMD Millipore	MAB4360	1:200	FCM
	Troponin I	Mouse IgG2b	EMD Millipore	MAB1691	1:200	ICC
	Troponin T	Mouse IgG1	Fisher	MS-295-P1	1:200	ICC
	VE-Cadherin	Rabbit IgG	Cell Signaling	2158	1:100	FCM
Secondary	Alexa 488, Goat anti-mouse IgG1		Life Technologies	A21121	1: 400 or 1:1000	ICC, FCM
	Alexa 594, Goat anti-rabbit IgG		Life Technologies	A11012	1: 400 or 1:1000	ICC, FCM
	Alexa 594, Goat anti-mouse IgG2b		Life Technologies	A21145	1: 400 or 1:1000	ICC, FCM
	Alexa 594, Goat anti-mouse IgM		Life Technologies	A21044	1: 400 or 1:1000	ICC, FCM
Conjugated	APC human CD31		Miltenyi Biotech	130092652	1:11	FCM
	BB515 Mouse Anti-Human CD140a (PDGFRα)		BD Biosciences	564594	1 : 40	FCM
	BV421 Mouse Anti-Human CD13		BD Biosciences	562596	1 : 20	FCM
	PE Mouse Anti-Human CD309 (KDR)		BD Biosciences	560494	1 : 10	FCM
	PE Mouse Anti-ISL1		BD Biosciences	562547	1:100	FCM
	PE Mouse Anti-Cardiac Troponin T		BD Biosciences	564767	1:100	FCM
	PE Mouse Anti-AKT (pS473)		BD Biosciences	560378	1 : 5	FCM
	PE/Cy7 anti-human CD172a/b (SIRPα/β)		Biolegend	323807	1 : 40	FCM

Table S3. Primers List for qRT-PCR.

Gene	Full name (other name)	Accession code	Primer
ANLN	Anillin, actin binding protein	NM_018685	Forward : TCCAAAGAAGATAAAAAGGGG<br>Reverse: CTGTGCGAACCAGCAACTT
ATP2A2	ATPase, Ca^{++} transporting, cardiac muscle, slow twitch 2	NM_170665	Forward :TCAGCAGGAACTTTGTCACC<br>Reverse: GGGCAAAGTGTATCGACAGG
AURKA	Aurora kinase A	NM_198433	Forward : TGGGTGGTCAGTACATGCTC<br>Reverse: TGCATCCGACCTTCAATCATTTC
AURKB	Aurora kinase B	NM_004217	Forward : CAGTGGGACACCCGACATC<br>Reverse: GTACACGTTTCCAAACTTGCC
BIRC5	Baculoviral IAP repeat containing 5	NM_001012271	Forward : CTTTCTCCGCAGTTTCCTCA<br>Reverse: TTGGTGAATTTTTGAAACTGGA
CASQ2	Calsequestrin 2	NM_001232	Forward: TTATGTTCAAGGACCTGGGC<br>Reverse: GCCTCTACTACCATGAGCCG
CCNB1	Cyclin B1	NM_031966	Forward: ACAGGTCTTCTTCTGCAGGG<br>Reverse: GAACCTGAGCCAGAACCTGA
GAPDH	Glyceraldehyde-3-phosphate dehydrogenase	NM_001256799	Forward : CTGGGCTACACTGAGCACC<br>Reverse: AAGTGGTCGTTGAGGGCAATG
HSP60	Heat shock 60kDa protein 1 (chaperonin) (HSPD1)	NM_002156	Forward: TTGACTGCCACAACCTGAAG<br>Reverse: CACCGTAAGCCTTTGGTCAT
HSP70	Heat shock 70kDa protein 4 (HSPA4)	NM_002154	Forward: TACCTGGCTTTTAGCTGCTG<br>Reverse: CGCTAATGAGTATAGCGACCG
HSP90	Heat shock protein 90kDa beta (Grp94), member 1 (HSP90B1)	NM_003299	Forward: GCTGACGATGAAGTTGATGTGG<br>Reverse: CATCCGTCCTTGATCCTTCTCTA
MKI67	Marker of proliferation Ki-67	NM_002417	Forward: TGACTTCCTTCCATTCTGAAGAC<br>Reverse: TGGGTCTGTTATTGATGAGCC
MYH6	Myosin, heavy chain 6, cardiac muscle, alpha (*α-MHC*)	NM_002471	Forward : CTTCTCCACCTTAGCCCTGG<br>Reverse: GCTGGCCCTTCAACTACAGA
MYH7	Myosin, heavy chain 7, cardiac muscle, beta (*β-MHC*)	NM_000257	Forward : CGCACCTTCTTCTCTTGCTC<br>Reverse: GAGGACAAGGTCAACACCCT
MYL2	Myosin, light chain 2, regulatory, cardiac, slow (MLC-2V),	NM_000432	Forward : CGTTCTTGTCAATGAAGCCA<br>Reverse: CAACGTGTTCTCCATGTTCG

MYL7	Myosin, light chain 7, regulatory (MYL-2A)	NM_021223	Forward : CTTGTAGTCGATGTTCCCCG<br>Reverse: TCAAGCAGCTTCTCCTGACC
PCNA	Proliferating cell nuclear antigen, transcript variant 2	NM_182649	Forward : TCAGGTACCTCAGTGCAAAAG<br>Reverse: TGCAAGTGGAGAACTTGGAA
PLK1	Polo-like kinase 1	NM_005030	Forward : AGTCGACCACCTCACCTGTC<br>Reverse: GCCCCTCACAGTCCTCAATA
RYR2	Ryanodine receptor 2, cardiac	NM_001035	Forward : CAAATCCTTCTGCTGCCAAG<br>Reverse: CGAAGACGAGATCCAGTTCC
SLC8A1	Solute carrier family 8 (sodium/calcium exchanger), member 1	NM_021097	Forward : CTGGAATTCGAGCTCTCCAC<br>Reverse: ACATCTGGAGCTCGAGGAAA
TNNI1	Troponin I type 1	NM_003281	Forward : AGCATCAGGCTCTTCAGCA<br>Reverse: ACAGTCTGCAGTCTACGGCG
TNNI3	Troponin I type 3, cardiac	NM_000363	Forward :CTCAAACTTTTTCTTGCGGC<br>Reverse: GTGAAGAAGGAGGACACCGA
TNNT2	Troponin T type 2, cardiac	NM_001001431	Forward : GCGGGTCTTGGAGACTTTCT<br>Reverse: TTCGACCTGCAGGAGAAGTT
TTN	Titin (*Connectin*)	NM_133378	Forward : GGGTTCGATTTTCCTCTTGA<br>Reverse: AGCCAACCTGAGTCTGGAAG

Note: primers were retrieved from open access websites (http://primerdepot.nci.nih.gov/ or http://pga.mgh.harvard.edu/primerbank/)

Movie S1. 2D-SG Culture at Differentiation Day 20

Movie S2. 3D-SG Culture at Differentiation Day 20

Movie S3. 3D-MG Culture at Differentiation Day 20

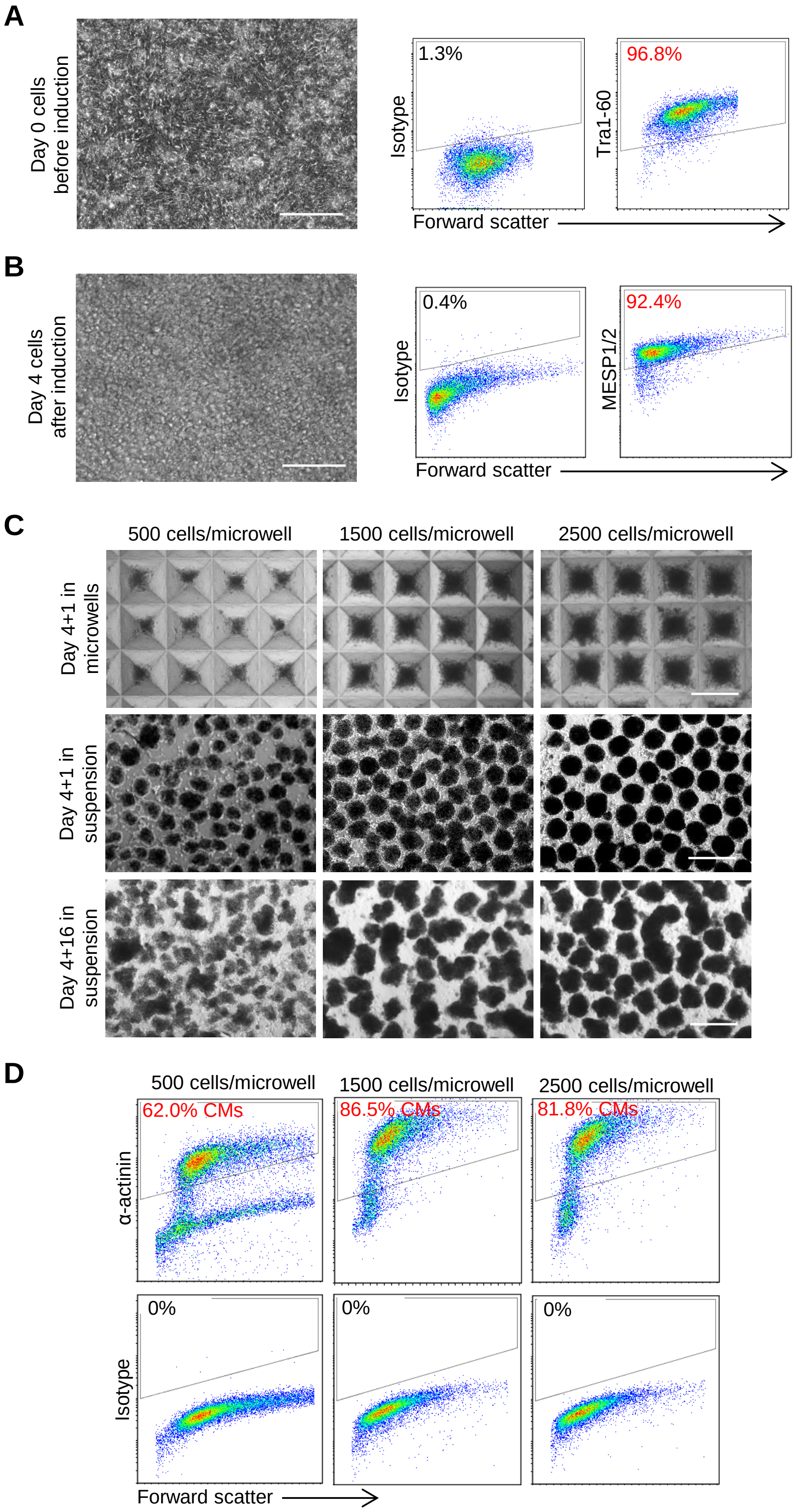

A
Day 0 cells before induction
1.3%
Isotype
96.8%
Tra1-60
Forward scatter
B
Day 4 cells after induction
0.4%
Isotype
92.4%
MESP1/2
Forward scatter
C
500 cells/microwell
1500 cells/microwell
2500 cells/microwell
Day 4+1 in microwells
Day 4+1 in suspension
Day 4+16 in suspension
D
500 cells/microwell
1500 cells/microwell
2500 cells/microwell
62.0% CMs
86.5% CMs
81.8% CMs
α-actinin
0%
0%
0%
Isotype
Forward scatter

Figure S1. **Microscale formation of progenitor cardiac spheres.** (**A**) hPSCs were induced to differentiate into CMs using activin A and BMP4. At day 0 before the induction, cells displayed as a layer of compact cells and ~97% of the cells expressed TRA1-60, a stem cell marker, as analyzed by flow cytometry. Scale bars = 200 μm. (**B**) At day 4, the cells lost the typical undifferentiated cell morphology but still displayed as a layer of compact cells. The majority of the day 4 cells (~95%) expressed MESP1/2, a marker for cardiac mesoderm, as analyzed by flow cytometry. Scale bars = 200 μm. (**C**) Morphologies of cells in a microwell plate seeded at the densities of 500, 1500 and 2500 cells/microwell at 24 h after the seeding (top panel) and additional 2 days in suspension cultures (bottom panel). Scale bar = 400 μm. (**D**) Flow cytometry analysis of α-actinin positive cells at differentiation day 20.

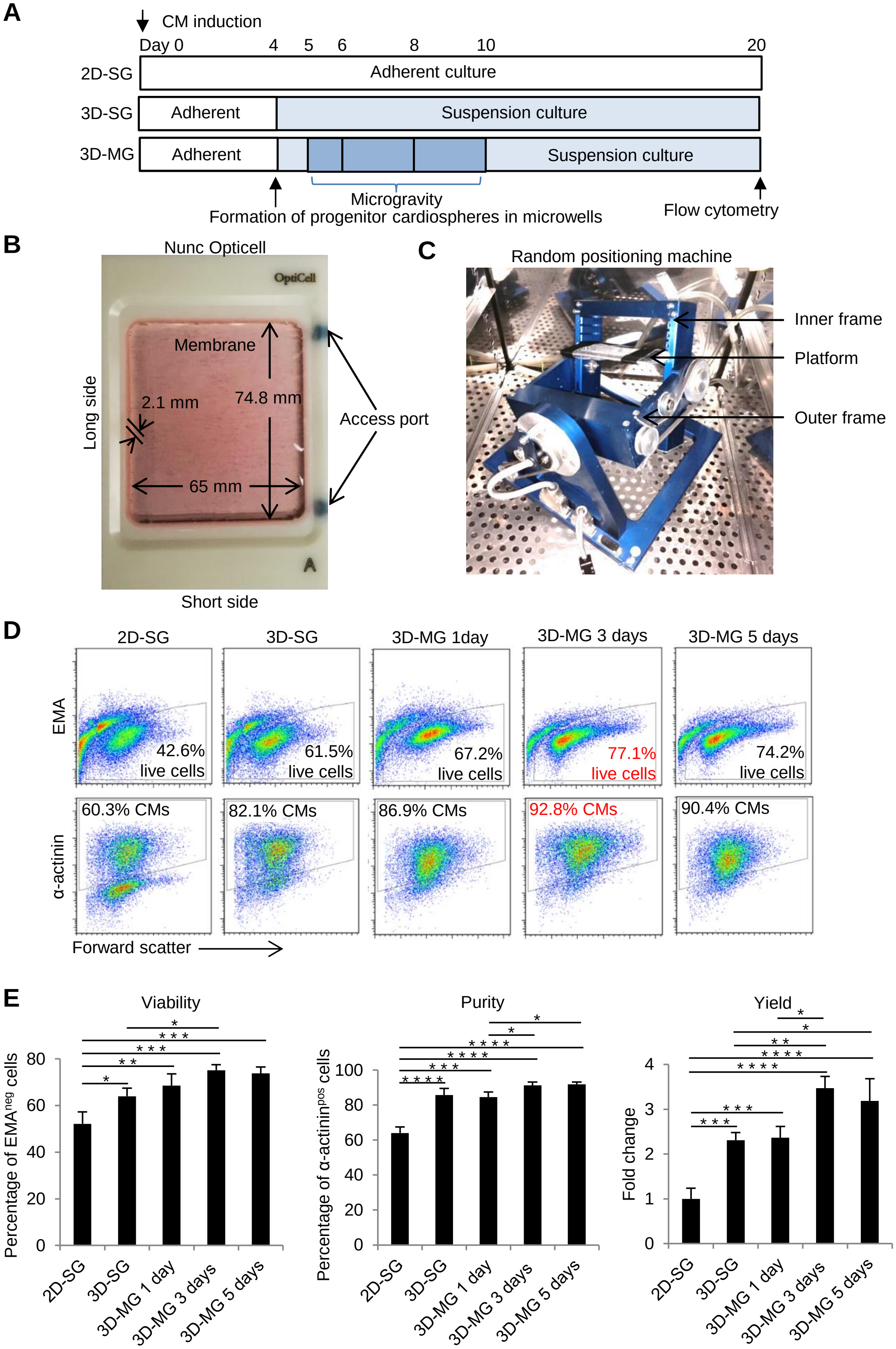
A
CM induction
Day 0
4
5
6
8
10
20
2D-SG
Adherent culture
3D-SG
Adherent
Suspension culture
3D-MG
Adherent
Suspension culture
Microgravity
Formation of progenitor cardiospheres in microwells
Flow cytometry
B
Nunc Opticell
Membrane
Long side
2.1 mm
74.8 mm
65 mm
Access port
Short side
C
Random positioning machine
Inner frame
Platform
Outer frame
D
2D-SG
3D-SG
3D-MG 1day
3D-MG 3 days
3D-MG 5 days
EMA
42.6% live cells
61.5% live cells
67.2% live cells
77.1% live cells
74.2% live cells
α-actinin
60.3% CMs
82.1% CMs
86.9% CMs
92.8% CMs
90.4% CMs
Forward scatter
E
Viability
Percentage of EMAneg cells
Purity
Percentage of α-actininpos cells
Yield
Fold change
2D-SG
3D-SG
3D-MG 1 day
3D-MG 3 days
3D-MG 5 days

Figure S2. **Effect of the duration of exposure to simulated microgravity on CM differentiation.** (**A**) Experimental design. hPSCs in 2D cultures were induced to differentiate into CMs at day 0 using a growth factor-guided method. At day 4, cells were dissociated and forced to aggregate into progenitor cardiac spheres. The spheres were cultured under simulated microgravity at days 5-6 (3D-MG 1 day), days 5-8 (3D-MG 3 days) or days 5-10 (3D-MG 5 days) and then maintained under standard gravity until day 20. Parallel 2D and 3D cultures under standard gravity throughout, designated as 2D-SG and 3D-SG cultures, respectively, were maintained. At day 20, cells were harvested and analyzed for cell viability, CM purity and cell yield. (**B**) OptiCell disk is a sealed cell culture disk formed between two optically clear, gas-permeable polystyrene membranes and contains two ports that allow access to the contents via a wide-bore needle attached with a syringe. The disk can be filled with 10- to 14-ml of medium and has 75 x 65 x 2 mm internal dimensions. (**C**) Random Positioning Machine (RPM) is composed of two motors that independently control the rotation of the outer and inner frames in random directions. OptiCell disks are mounted at the center of the platform attached to the inner frame. (**D**) Representative flow cytometry analysis. Cell viability was analyzed by EMA staining and EMA negative cells were identified as live cells. Purity of CMs was analyzed by intracellular staining of α-actinin, a CM-associated marker. (**E**) Summary of cell viability, CM purity, and cell yield at differentiation day 20 from IMR90-hiPSCs exposed to simulated microgravity for 1, 3 or 5 days. Data are presented as mean ± SD of 3 biological samples for each culture condition. *, $P<0.05$; **, $P<0.01$; ***, $P<0.001$, ****, $P<0.0001$.

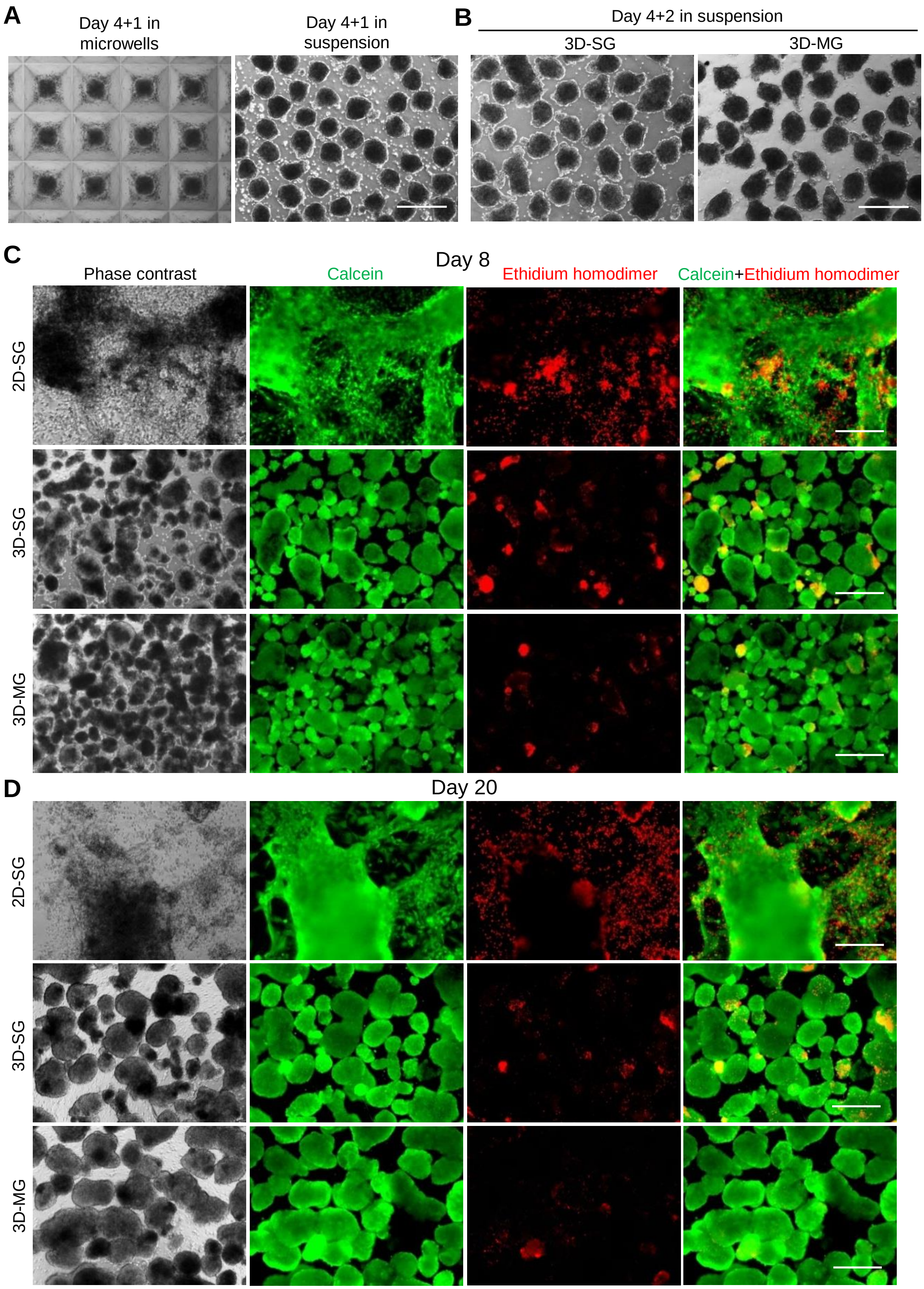
A
Day 4+1 in microwells
Day 4+1 in suspension
B
Day 4+2 in suspension
3D-SG
3D-MG
C
Day 8
Phase contrast
Calcein
Ethidium homodimer
Calcein+Ethidium homodimer
2D-SG
3D-SG
3D-MG
D
Day 20
2D-SG
3D-SG
3D-MG

Figure S3. Morphologies and viability of CM differentiation cultures. (**A**) Formation of progenitor cardiac spheres in microwells of an AggreWell 400 plate. Approximately 1500 cells/microwell were seeded into an 8-well AggreWell 400 plate by plating 1.8×10^6 to each well. The plate was gently centrifuged to capture the cells in the microwells. After 24 h, spheres were transferred to suspension cultures. Scale bar = 400 μm. (**B**) Morphologies of progenitor cardiac spheres in suspension cultured under standard gravity (3D-SG) or microgravity (3D-MG). Live/Dead staining of intact cultures at day 8 (**C**) and day 20 (**D**) showing viable cells (green) and dead cells (red) detected by calcein and ethidium homodimer, respectively. Scale bar = 400 μm.

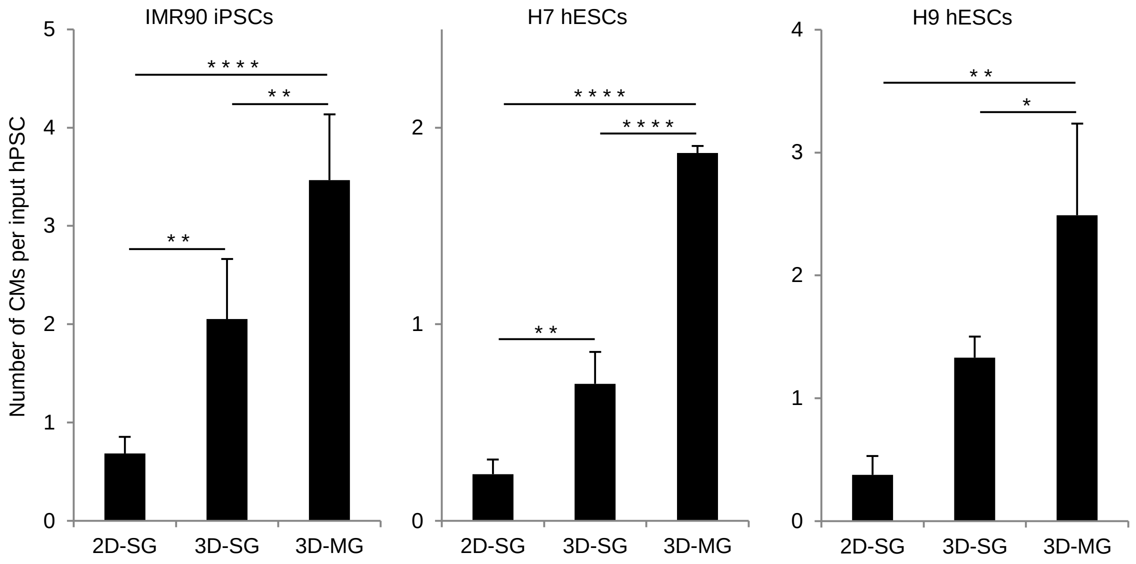

Figure S4. **Simulated microgravity and 3D culture increase CM yield.** Undifferentiated hPSCs were seeded at 2 x10^5 cells/cm^2 and induced for CM differentiation. At differentiation day 20, cultures derived from IMR90 iPSCs, H7 hESCs and H9 hESCs under 2D-SG, 3D-SG and 3D-MG conditions were analyzed for cell number, cell viability, and CM purity. For each culture, the number of viable CMs was calculated by multiplying the total number of cells, the percentage of live cells (EMA^{neg} cells) and the percentage of live CMs (α-$actinin^{pos}$ cells). Further, the number of CMs derived from one hPSC was calculated by dividing the number of viable CMs by the number of input hPSCs and presented as mean ± STD. n = 5 for IMR90 iPSCs; n =3 for H7 hESCs and H9 hESCs. *, P<0.05; **, P<0.01; ****, P<0.0001.

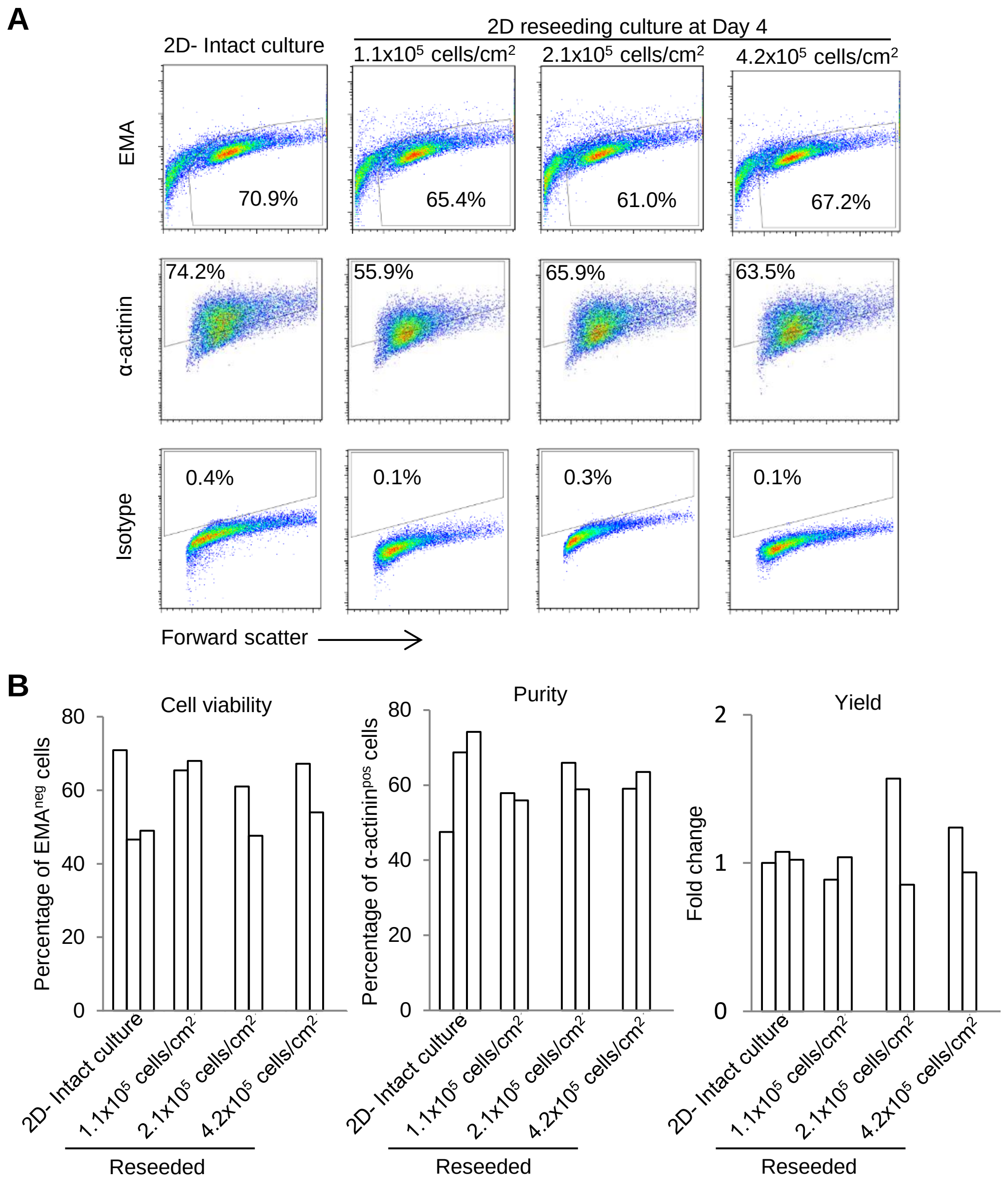

Figure S5. **Effect of replating day 4 cells in 2D culture on CM differentiation.** (**A**) Representative flow cytometry analysis at differentiation day 20. Day 4 cells were dissociated and re-seeded at 4-fold diluted (1.1x10^5 cells/cm^2), 2-fold diluted (2.1x10^5 cells/cm^2) and undiluted (4.2x10^5 cells/cm^2) densities and CM differentiation efficiency was compared at day 20 with parallel intact cultures. (**B**) Summary of cell viability, CM purity and yield. Cell yield was calculated based on final viable CMs generated from each input undifferentiated stem cell. Intact 2D-SG cultures (n = 3) and replated 2D cultures (n = 2 for each seeding density) were analyzed.

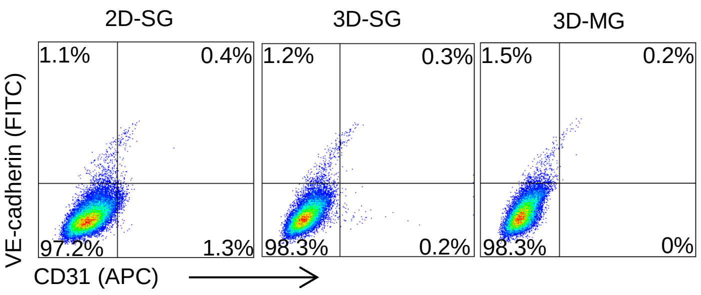

Figure S6. Detection of endothelial cells in CM differentiation cultures. Differentiated cells in 2D-SG, 3D-SG and 3D-MG conditions at day 20 were analyzed for endothelial cell differentiation by flow cytometry analysis of CD31 and VE-cadherin. Very few CD31^{pos} and VE-cadherin^{pos} cells were detected in all cultures (<2% of the total cell population).

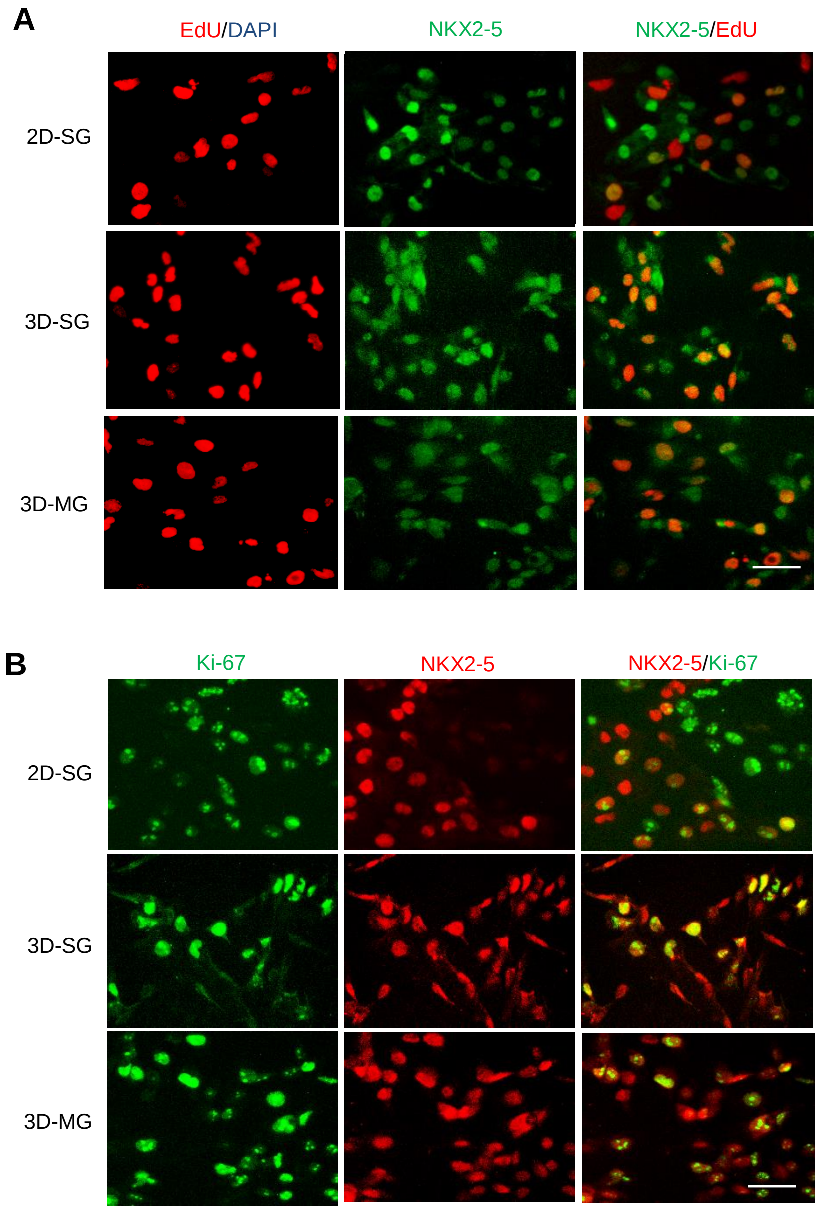

Figure S7. Detection of proliferative cardiac cells in CM differentiation cultures. Differentiated cells in 2D-SG, 3D-SG and 3D-MG conditions at day 8 were dissociated and replated. Cells were analyzed by immunocytochemical analysis 2 day after plating. **(A)** Representative images and summary of cells at differentiation day 10 that were co-stained with EdU and an antibody against NKX2-5, a marker for cardiac progenitors. (**B**) Similar analysis to (A) except that Ki-67 instead of EdU was co-stained with NKX2-5. Scale bars = 100 µm.

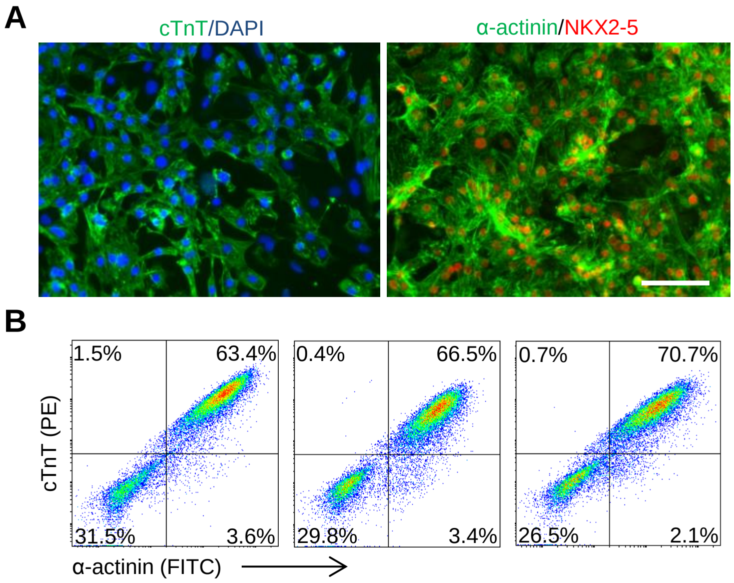

Figure S8. Detection of CMs in differentiation cultures. (**A**) On day 20 post-induction with activin A and BMP4, cells were dissociated, replated and subjected to immunocytochemical analysis. The majority of the cells were positive for cTnT, α-actinin and NKX2-5. Note that almost all α-actinin^{pos} cells were also positive for NKX2-5, a cardiac specific transcription factor. (**B**) Flow cytometry analysis of differentiation day 20 showing that ~95-97% α-actinin^{pos} cells were also positive for cTnT, a cardiac specific maker.
